# Seascape Genomics and Phylogeography of the Sailfish (*Istiophorus platypterus*)

**DOI:** 10.1093/gbe/evad042

**Published:** 2023-03-13

**Authors:** Bruno Lopes da Silva Ferrette, Raphael T F Coimbra, Sven Winter, Menno J De Jong, Samuel Mackey Williams, Rui Coelho, Daniela Rosa, Matheus Marcos Rotundo, Freddy Arocha, Bruno Leite Mourato, Fernando Fernandes Mendonça, Axel Janke

**Affiliations:** Senckenberg Biodiversity and Climate Research Centre, Frankfurt am Main, Germany; LOEWE Centre for Translational Biodiversity Genomics, Frankfurt am Main, Germany; Conservation Genetics Laboratory, Santa Cecília University, Santos, Brazil; Senckenberg Biodiversity and Climate Research Centre, Frankfurt am Main, Germany; Institute for Ecology, Evolution and Diversity, Goethe University, Frankfurt am Main, Germany; Senckenberg Biodiversity and Climate Research Centre, Frankfurt am Main, Germany; Research Institute of Wildlife Ecology, Vetmeduni Vienna, Vienna, Austria; Senckenberg Biodiversity and Climate Research Centre, Frankfurt am Main, Germany; School of Biological Sciences, The University of Queensland, Queensland, Australia; Instituto Português do Mar e da Atmosfera (IPMA), Olhão, Portugal; Centro de Ciências do Mar (CCMAR), Universidade do Algarve, Faro, Portugal; Instituto Português do Mar e da Atmosfera (IPMA), Olhão, Portugal; Centro de Ciências do Mar (CCMAR), Universidade do Algarve, Faro, Portugal; Acervo Zoológico (AZUSC), Universidade Santa Cecília (UNISANTA), Santos, Brazil; Instituto Oceanográfico de Venezuela, Universidad de Oriente (UDO), Camaná, Venezuela; Laboratório de Ciências da Pesca (LABPESCA), Instituto do Mar (IMar), Universidade Federal de São Paulo (UNIFESP), Campus Baixada Santista, Santos, Brazil; Laboratório de Genética Pesqueira e Conservação (GenPesC), Instituto do Mar (IMar), Universidade Federal de São Paulo (UNIFESP), Campus Baixada Santista, Santos, Brazil; Senckenberg Biodiversity and Climate Research Centre, Frankfurt am Main, Germany; LOEWE Centre for Translational Biodiversity Genomics, Frankfurt am Main, Germany; Institute for Ecology, Evolution and Diversity, Goethe University, Frankfurt am Main, Germany

**Keywords:** reference genome assembly, whole-genome sequencing, genome-wide heterozygosity, demographic history, fisheries management units

## Abstract

Permeable phylogeographic barriers characterize the vast open ocean, boosting gene flow and counteracting population differentiation and speciation of widely distributed and migratory species. However, many widely distributed species consists of distinct populations throughout their distribution, evidencing that our understanding of how the marine environment triggers population and species divergence are insufficient. The sailfish is a circumtropical and highly migratory billfish that inhabits warm and productive areas. Despite its ecological and socioeconomic importance as a predator and fishery resource, the species is threatened by overfishing, requiring innovative approaches to improve their management and conservation status. Thus, we presented a novel high-quality reference genome for the species and applied a seascape genomics approach to understand how marine environmental features may promote local adaptation and how it affects gene flow between populations. We delimit two populations between the Atlantic and Indo-Western Pacific oceans and detect outlier loci correlated with sea surface temperature, salinity, oxygen, and chlorophyll concentrations. However, the most significant explanatory factor that explains the differences between populations was isolation by distance. Despite recent population drops, the sailfish populations are not inbred. For billfishes in general, genome-wide heterozygosity was found to be relatively low compared to other marine fishes, evidencing the need to counteract overfishing effects. In addition, in a climate change scenario, management agencies must implement state-of-the-art sequencing methods, consider our findings in their management plans, and monitor genome-wide heterozygosity over time to improve sustainable fisheries and the long-term viability of its populations.

**Significance**

Highly migratory and widespread marine species usually present high gene flow levels between populations. However, the sailfish shows two highly divergent populations that lack gene flow between the Atlantic and Indo-Western Pacific oceans. These populations presumably evolved during the Pleistocene glacial cycles and were isolated for prolonged periods, but not sufficiently long for speciation to occur. The sailfish has lower genetic diversity than other marine fishes, although no recent inbreeding was detected. These results will improve the fisheries management for the sailfish, which requires establishing new and collaborative management plans. Besides, the results will enable comparative genomic studies and aid the assembly of high-quality reference genomes of closely related species.

## Introduction

The open ocean lacks clear phylogeographic barriers that would prevent migration between populations. As a result, widely distributed marine species can sustain a circumtropical distribution counteracting local adaptation, population differentiation, and speciation. Yet, in practice, many marine species present deep evolutionary lineages that did not speciate, but have either confined or extensive distribution ranges with pronounced population differentiation between ocean basins. However, the factors underlying these speciation or population dynamics are not entirely understood, although habitat partitioning rather than allopatric isolation may have conducted these evolutionary forces (Bowen et al. 2016; Gaither et al. 2016; Reid et al. 2016).

Among these species, the sailfish (*Istiophorus platypterus*) is a highly migratory, circumtropical species characterized by its discernible sail-like dorsal fin, and like other billfishes (Istiophoridae and Xiphiidae), it inhabits a narrow range of environmental features preferring moderately warm and productive regions (Reygondeau et al. 2012; Thoya et al. 2022). It is a valuable fishery resource and sport fish providing food security and income for developing and developed countries (Kadagi et al. 2020; Wosnick et al. 2021). Given its socioeconomic relevance, the sailfish's stocks are managed in each ocean basin by different regional fisheries management organizations (RFMOs). However, their fishery management plans do not consider previous genetic population studies with traditional molecular markers that were able to delimit differentiated management units between and within the oceans basins, which contradicts the current RFMOs fishery stocks’ delimitation, undermining the sustainable management and conservation efforts for this important fishery resource (Hoolihan et al. 2004; Graves and McDowell 2015; Lu et al. 2015; Rubio-Castro et al. 2016; Ferrette et al. 2021). Furthermore, the genus *Istiophorus* is formed by two highly divergent evolutionary lineages that could indicate the presence of cryptic species that need to have their taxonomic status clarified; otherwise, they can also compromise sustainable fisheries management for the species (Ferrette et al. 2021).

**Fig. 1.**
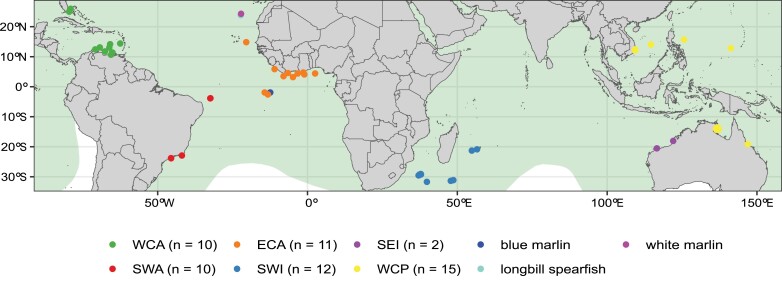
Sampling locations of sailfish (*I. platypterus*) specimens and the species known distribution (green), blue marlin (*M. nigricans*), white marlin (*K. albida*), and shortbill spearfish (*T. angustirostris*). Numbers show the sample size in each region.

As a result, recent stock assessments suggest a high potential for overfishing and shrinking in its distribution ranges (Worm and Tittensor 2011; Punt et al. 2015; Sharma et al. 2018). In addition, the species was recently assessed as “vulnerable” by the International Union for Conservation of Nature (IUCN) Red List of Threatened Species (Collette et al. 2022a), which makes the sailfish's management even more relevant. A better understanding of its range distribution dynamics and population structure delimitation is crucial for effective stock management. Currently, most fishery stocks are fully exploited, overexploited, depleted, or need recovery (McCauley et al. 2015; Pauly and Zeller 2016), and overfishing is a major threat that can reduce biomass and genetic diversity (Pinsky and Palumbi 2014). However, implementing effective fisheries management has decreased mortality rates for tunas and billfishes (Juan-Jordá et al. 2022). Therefore, improving management measures is required to rebuild fishery stocks, ensure a sustainable fishery, and decrease extinction risks of fishery resources.

Next-generation sequencing (NGS) and cost reduction have given rise to the genomics era, whereby genome-wide approaches rapidly replace traditional genetic marker approaches (Brandies et al. 2019). High-quality and complete reference genome assemblies are essential for applying genomics to wildlife management and conservation (Formenti et al. 2022). However, there is a lack of reference genomes available for non-model organisms, compromising their application to improve our knowledge of species’ biology and extinction risks, limiting our understanding of species’ taxonomy, evolutionary processes, and adaptive potential (Rhie et al. 2021). Population genomics applies a novel scale and density of genome-wide markers using NGS and high-capacity computational technologies, providing more accuracy in detecting genomic regions associated with traits or evolutionary processes such as fitness, phenotypes, and selection, allowing the evaluation of genetic variation across the entire genome to understand how different habitats or environmental features influence adaptive and neutral genomic variation within and among populations and species, improving wildlife and fisheries management (Liggins et al. 2019; Hohenlohe et al. 2021).

The lack of genome-wide data for the sailfish compromises our understanding of its genome-wide heterozygosity, fishery stocks delimitation, and resilience to overfishing and climate change (Rossetto et al. 2021). Therefore, we report a novel, high-quality de novo genome assembly for the species and supply the first state-of-the-art whole-genome resequencing data set comprising samples across the Atlantic (ATL), Indian, and Pacific oceans (fig. 1) (Collette et al. 2022a), inserting the management of the sailfish and its relatives into the genomics era. Furthermore, we applied a seascape genomics approach (Selkoe et al. 2016) to evaluate the sailfish's genomic population structure and how contemporary environmental change trends and anthropogenic pressures may trigger ecological and evolutionary consequences for its sustainable management and conservation efforts.

## Results

### Genome Assembly

**Fig. 2.**
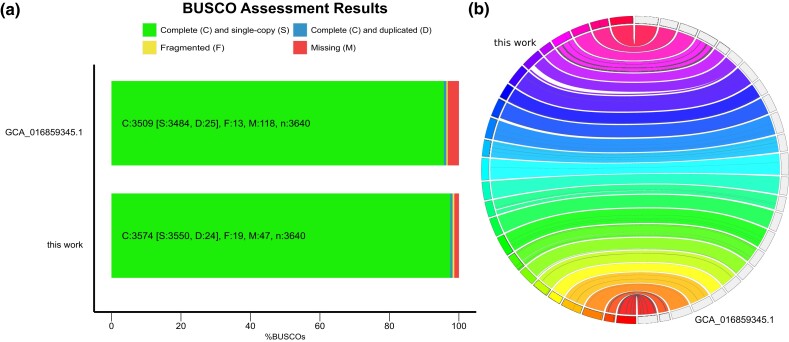
Genome assembly completeness. (*a*) BUSCO assessments results for gene set completeness between our newly generated chromosome-level assembly from an ATL sailfish and the Indo-Pacific sailfish genome under GenBank assembly accession: GCA_016859345.1. (*b*) Synteny between our newly generated chromosome-level assembly from an ATL sailfish and the Indo-Pacific sailfish genome.

The final chromosome-level de novo assembled genome has a length of 619.037 mega bases (Mb) divided into 404 scaffolds with contig N50 of 26.28 Mb, L50 of 11, and guanine-cytosine (GC) content of 40.87%. The largest 24 contigs (>1 Mb) make up over 99% of the total assembly length (supplementary table S1, Supplementary Material online). Repetitive regions span 19.66% of the total assembly length. DNA transposons were the most common repeats (6.24%) of which short interspersed elements (SINEs), long interspersed nuclear elements (LINEs), and long terminal repeats (LTRs) elements accounted for 2.97% (supplementary table S2, Supplementary Material online). The rest of the repetitive regions were low complexity, satellites, simple repeats, ribosomal RNAs (rRNAs), small conditional RNAs (scRNAs), small nuclear RNAs (snRNAs), and transfer RNAs (tRNAs). The genome assembly completeness analysis found 98.3% complete Benchmarking Universal Single-Copy Orthologs (BUSCO) genes (97.6% complete and single copy) and only 1.2% missing BUSCOs, which suggests that the assembly holds most of the coding regions of the genome (fig. 2*a*). Furthermore, Merqury estimated an assembly completeness of 95.86% with an assembly consensus quality value of 29.48, standing for an error rate of 0.11%. A high mapping rate of >99% for short reads and 95.66% long reads without contamination confirmed the assembly's completeness (supplementary fig. S6, Supplementary Material online). Furthermore, our new sailfish genome assembly displayed high synteny and consistency with only a few misassembled regions and only a tiny fraction of chromosomal rearrangements compared to the assembly of Wu et al. (2021) (fig. 2*b*). Short reads’ mapping statistics per sample are shown in supplementary table S3, Supplementary Material online.

### Genomic Population Structure

**Fig. 3.**
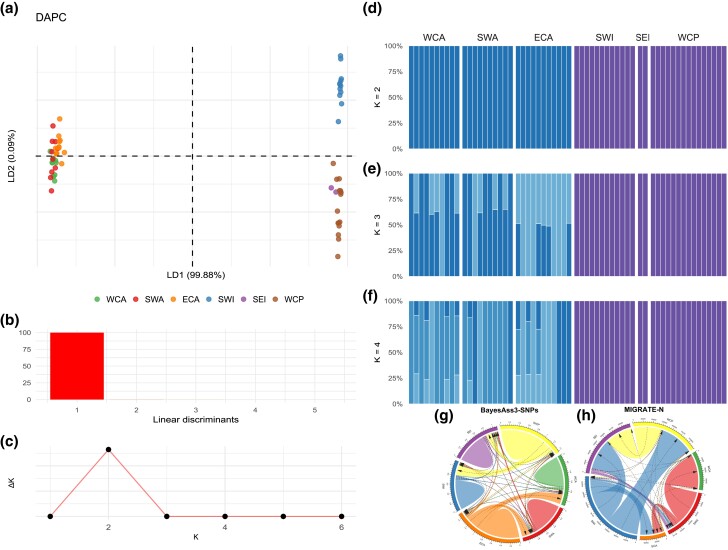
Clustering and admixture analysis. (*a*) DAPC from DAs 1 and 2. (*b*) Eigenvalues for the DAs. (*c*) Evanno's Δ*K* method. (*d*–*f*) Individual ancestral admixture proportions estimations from *K* = 2–4. (*g*) Short-term immigration rates between sampled regions using unlinked genome-wide SNPs. (*h*) Long-term immigration rates between sampled regions using the mitogenomes. Numbers indicate the migration rates between regions.

Our final dataset after single-nucleotide polymorphism (SNP) calling filtering, and linkage disequilibrium (LD) pruning comprised 428,675 unlinked sites. The discriminant analysis of principal components (DAPC) exhibited two highly divergent clusters between the ATL and Indo-Western Pacific (IDWP) oceans with linear discriminants of 99.88% in the first axis (fig. 3*a* and *b*). It also showed a possible substructuring between populations in the Southwest Indian (SWI) and Western Central Pacific (WCP) oceans. However, the ancestral admixture coefficients from *K* = 2 to *K* = 4, showed no evidence for substructuring between these populations. The Δ*K* method yielded the best value of *K* = 2 (fig. 3*c*–*e*). Supplementary Figures S7–9, Supplementary Material online show all admixture coefficient proportions from *K* = 2 to *K* = 6 between and within each ocean basin. As a subproduct, we provided a subset of 200 highly informative SNPs for population analysis to nurture future fishery stock assessments for the sailfish (supplementary fig. S10 and S11, Supplementary Material online).

In addition, we found that most short-term migration occurred within each ocean basin, not between them, reinforcing the existence of two populations. As much as in the ATL as IDWP, migration occurs westward from East Central Atlantic (ECA) toward Western Central Atlantic (WCA) (*m* = 0.0196 ± 0.0184) and Southwest Atlantic (SWA) (*m* = 0.0195 ± 0.0185) and from WCP toward SEI (*m* = 0.0159 ± 0.0152) and Southwest Indian (SWI) (*m* = 0.0159 ± 0.0152), respectively (fig. 3*g*).

**Fig. 4.**
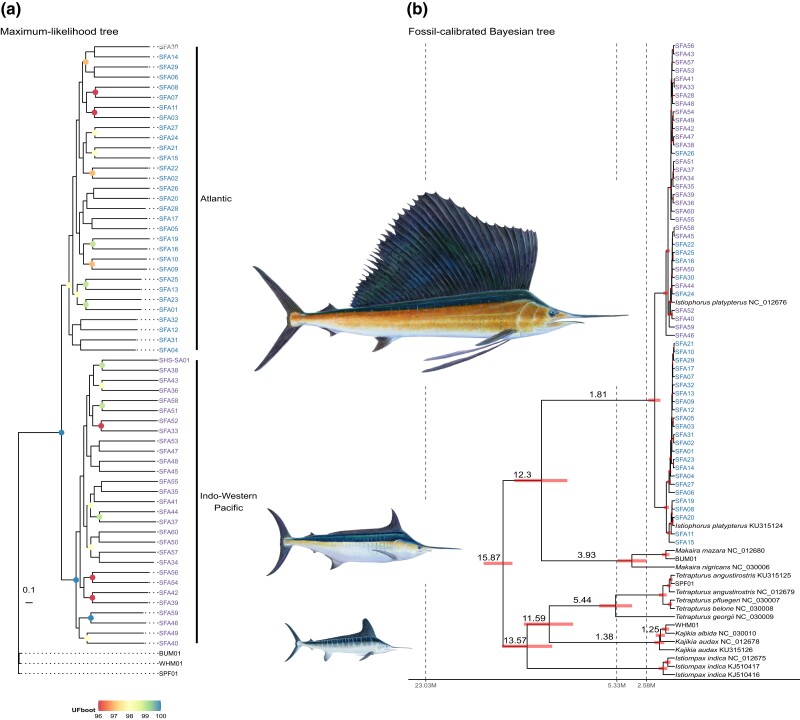
Phylogenetic inference. (*a*) ML phylogenetic tree estimated from genome-wide SNPs. Node color gradient displays the ultrafast bootstraps approximation (UFBoot) values from 96 to 100. (*b*) Fossil-calibrated Bayesian phylogenetic inference estimated from the mitogenomes. Node numbers show the age estimation; red bars indicate the 95% highest posterior density (HPD) intervals. Color names display the respective ocean basin where individuals belong: blue for the ATL Ocean and purple for the Indo-Pacific. Dashed lines represent the lower boundaries between the Cenozoic epochs, Pleistocene (2.58 M), Pliocene (5.33 M), and Miocene (23.03 M), respectively. SFA, *I. platypterus*; BUM, *M. nigricans*; WHM, *K. albida*; SPF, *T. angustirostris*. Names in blue are from the ATL lineage, while purple names are for the Indo-Pacific lineage.

The genomic maximum likelihood (ML) phylogeny shows that the genus *Istiophorus* is monophyletic and forms two highly supported evolutionary lineages composed of specimens from each ocean basin (98% and 100%, respectively) (fig. 4*a*). Nevertheless, the raw genetic distance between these lineages (*D_xy_* = 0.264%) and the *F*_ST_ (0.03, *P* < 0.001) are too low for them to be considered distinct species (supplementary fig. S12 and S20, Supplementary Material online). However, these results showed the need to treat these divergent populations as distinct fisheries management units by their respective RFMOs.

### Seascape Genomics

The three genome scans reported different numbers of outlier loci. Genome-wide differentiation scan (GWDS) reported 1,147 outliers, OutFLANK 567, and *pcadapt* 700. However, only 82 outlier loci were detected simultaneously by the three methods (supplementary fig. S13, Supplementary Material online). According to the forward selection, the redundancy analysis (RDA) is best modeled using only four environmental variables: mean dissolved oxygen, chlorophyll and sea surface salinity concentrations, and sea surface temperature (supplementary tables S4 and S5, Supplementary Material online). The RDA full models detected significant correlation between the genetic differentiation and the remaining variables (*P* < 0.001), and axis 1 was the most significant in explaining the data correlation (60.07% and 59.84%, respectively), even though the four environmental variables were also significant (*P* < 0.001) (supplementary tables S6–11, Supplementary Material online).

**Fig. 5.**
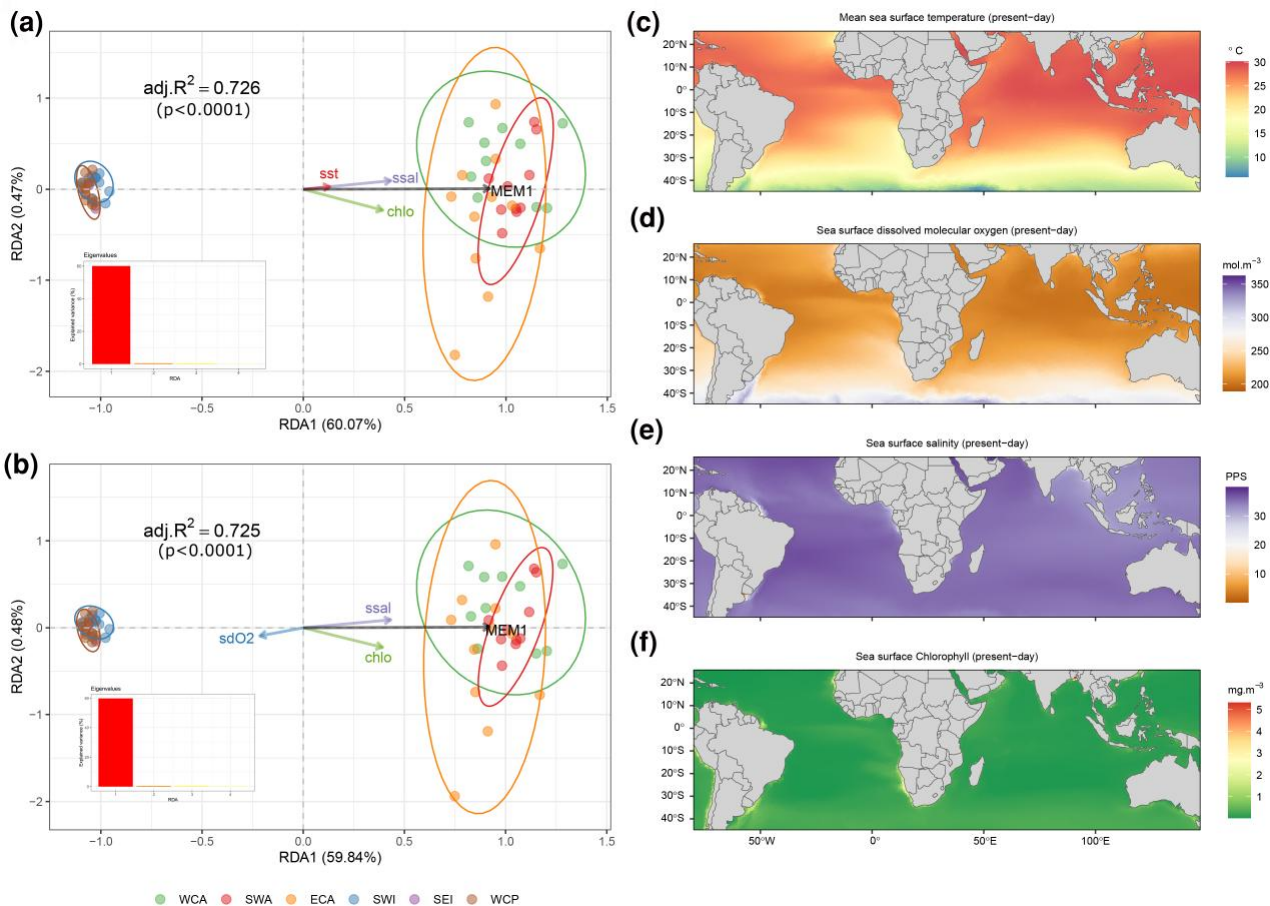
Redundancy Analysis (RDA). (*a* and *b*) RDAs displaying the correlation between variables, axes numbers show the percentage of the explanation of variance. (*c*–*f*) chlo, mean chlorophyll concentration; sst, mean sea surface temperature; ssal, mean sea surface salinity; sdO_2_, mean concentration of surface dissolved molecular oxygen. MEM, Moran's Eigenvector Maps. adj.*R*^2^, the adjusted *R*^2^ adjusted for the number of explanatory variables and *P*-values.

According to the RDAs, geographical distance plays a key role in explaining the differences between both populations, given that MEM1 strongly drives the variation between populations. Nevertheless, the RDAs showed that ATL outliers positively correlate to the mean chlorophyll concentration, mean sea surface temperature, and mean sea surface salinity but negatively relate to the mean dissolved oxygen. However, IDWP is positively correlated to dissolved oxygen and negatively correlated to the other variables (fig. 5*a* and *b*). Figure 5*c*–*f* exhibits the variation of the most significant environmental features.

### Demographic History, Heterozygosity, and Inbreeding

**Fig. 6.**
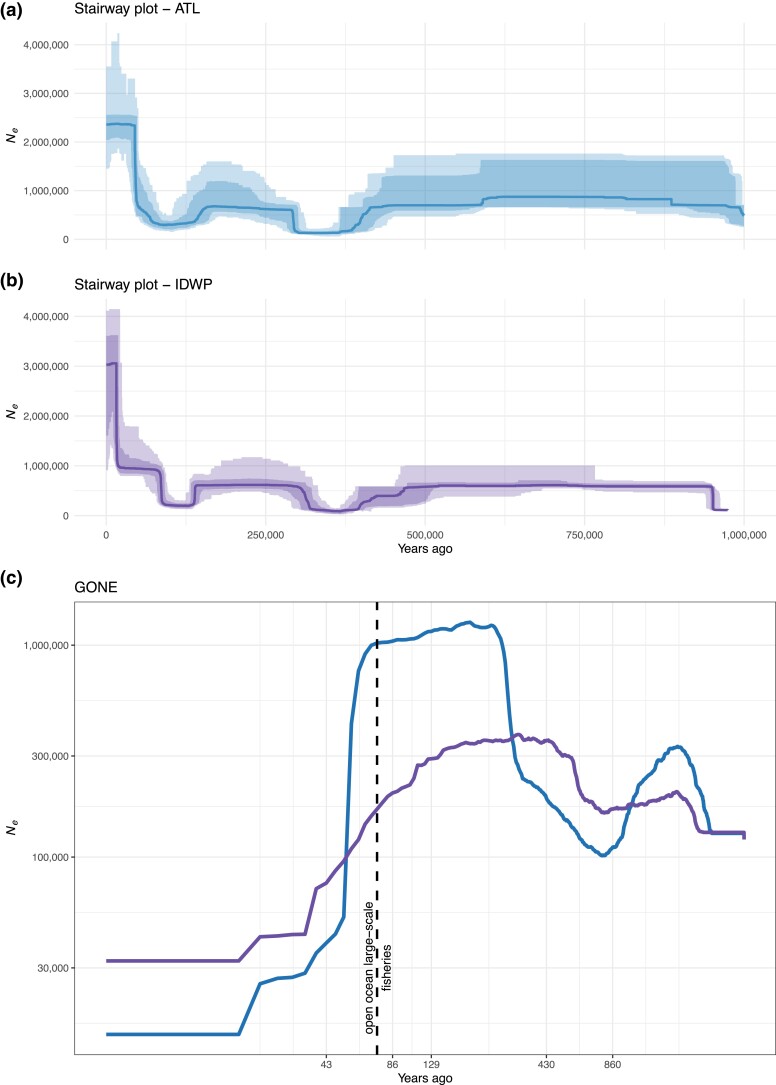
Demographic history. (*a*) Stairway plot from the ATL Ocean. (*b*) Stairway plot from the IDWP. (*c*) GONE. *N_e_*, effective population size. ATL population: blue; IDWP population: purple.

Pairwise sequentially Markovian coalescent (PSMC) showed an increase in the effective population size (*N_e_*) for most billfishes during the Middle Pleistocene (774–129 thousand years ago [Kya]), except for the swordfish that delivered a smooth increase in *N_e_* during the Late Pleistocene (129–11.7 Kya) (supplementary fig. S15, Supplementary Material online), but in general, the family Istiophoridae exhibited a *N_e_* decreasing trend. Furthermore, Stairway plots displayed a reduction trend in *N_e_* for both populations with two intense bottleneck events during the Pleistocene glaciation cycles (fig. 6*a* and *b*). The genetic optimization for *N_e_* estimation (GONE) showed the most recent demographic history trajectories for each population; both populations displayed an abrupt *N_e_* plummet around 80 years ago, in the 1950s when the open ocean large-scale industrial fisheries began and had the highest fishery catch statistics reports (Crespo and Dunn 2017). ATL suffered a 60-fold *N_e_*’s decrease, while IDWP had a 10-fold reduction (fig. 6*c*).

**Fig. 7.**
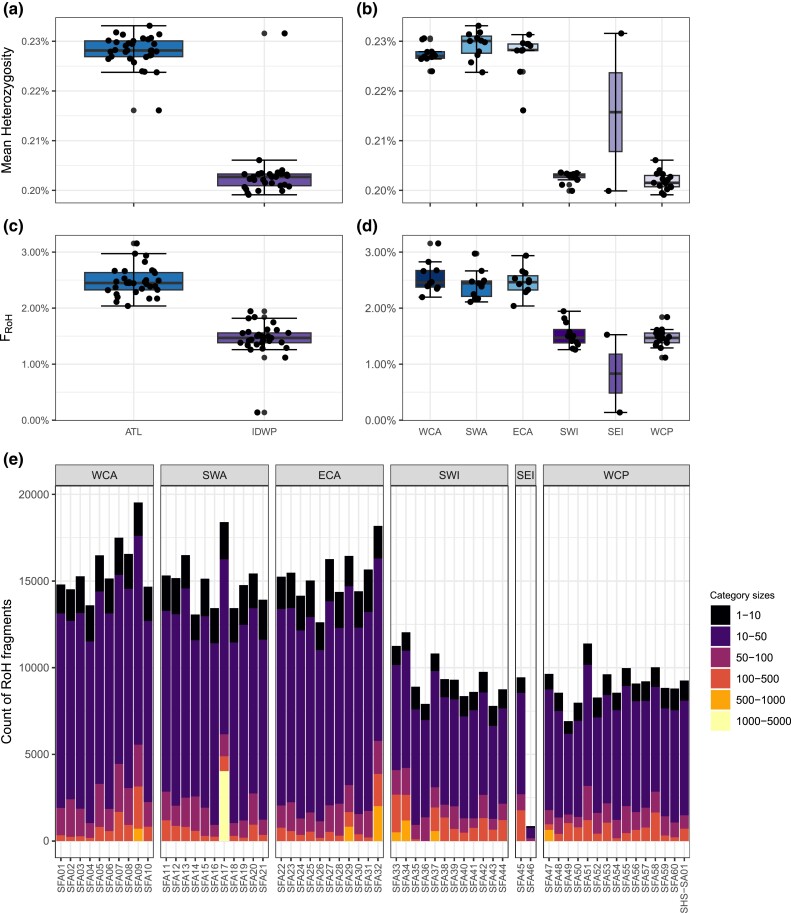
Heterozygosity and inbreeding. (*a*) Box plots of heterozygosity per population. (*b*) Box plots of heterozygosity per sampled region. (*c*) Box plots of the proportion of the genome covered by RoH (*F*_RoH_) per population. (*d*) Box plots of the proportion of the genome covered by RoH (*F*_RoH_) per sampled region. (*e*) Gradients of the length distribution of RoHs fragments per individual.

The average genome-wide heterozygosity was 0.216%, and it was higher in ATL (0.228%) than in IDWP (0.203%) (fig. 7*a*). Heterozygosity ranged from 0.202% in WCP to 0.229% in WCA (fig. 7*b*). The average of runs of homozygosity (RoH)-based inbreeding coefficients (*F*_RoH_) for the sailfish was 2.18%, with a higher inbreeding in ATL individuals (2.48%) than in IDWP (1.44%) (fig. 7*c*). *F*_RoH_ ranged from 2.76% on WCA to 0.92% in SEI (fig. 7*d*). Sailfish individuals had an average of 691.09 RoHs, most of which were short fragments ranging from 1 to 50 Kbp (fig. 7*e*), evidencing no recent inbreeding in both populations. Supplementary Table S12 and supplementary figures S16–18, Supplementary Material online show the genome-wide heterozygosity and RoHs summary statistics.

### Mitogenomic Phylogeography

**Fig. 8.**
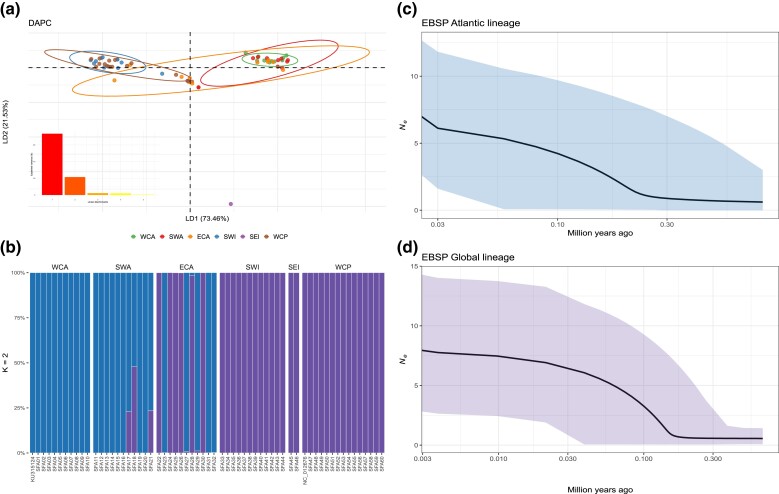
Mitogenomic analyses. (*a*) DAPC. (*b*) Posterior probability of cluster membership for sampled individuals with *K* = 2 (not admixture coefficients). (*c* and *d*) eBSP. *N_e_*, effective population sizes.

The sailfish's mitogenomes showed a mean length of 16,523 base pairs (bp), ranging from 16,519 to 16,539 bp with nucleotide frequencies of adenine (A: 27.4%), cytosine (C: 30.5%), guanine (G: 16.4%), and thymine (T: 25.8%). Mitogenomic outcomes showed differences from genome-wide nuclear data; DAPC displayed overlapping clusters with minor differences between ATL and IDWP (fig. 8*a* and *b*). The hierarchical analysis of molecular variance (AMOVA) scenario I rejected the null hypothesis of the absence of genetic population structure (supplementary table S13, Supplementary Material online). Scenario II showed the higher structuring scenario is between the two ocean basins resulting in a Φ_ST_ = 0.558 (*P* < 0.001). The pairwise Φ_ST_ evidenced that structuring between the populations is higher from a mitogenomic perspective because the most significant values are between the ATL and IDWP regions (supplementary fig. S20, Supplementary Material online).

The topology of the fossil-calibrated tree showed that all extant billfishes diverged during the Middle Miocene, although the modern lineages diverged at the beginning of the Pliocene around 5.33 Ma. In addition, the sailfish lineages diverged at 1.81 Ma during the Early Pleistocene (2.58–1.80 Ma) (fig. 5*b*; supplementary fig. S18, Supplementary Material online). One of the lineages is comprised of only ATL individuals, the other comprises individuals from all three ocean basins (fig. 4*b*; supplementary figs. S21 and S22, Supplementary Material online). In addition, both lineages display an increase in *N_e_* after 300 Kya (fig. 8*c* and *d*). The migration rates show a stepping-stone dispersal from the IDWP toward the ATL Ocean, in which SWI connects with ECA and SWA (fig. 3*h*).

## Discussion

### Genome Assembly

Our new chromosome-level genome assembly supplies a novel and improved high-quality reference genome for the sailfish. In comparison to the recently published assembly from Wu et al. (2021), our assembly is slightly larger (619.036 Mb vs. 614.147 Mb), more contiguous on a contig level (N50 of 23.35 Mb vs. 11.85 Mb) and has higher BUSCO genes completeness (98.3% vs. 95.3%) with fewer “Ns” per 100 Kbp (7.59 vs. 13.43). These assemblies will significantly improve the sailfish's fishery stocks assessment allowing cost-efficient population genomics and evolutionary inference studies using low-coverage whole-genome resequencing data and, in this sense, providing more accurate assessments (Attard et al. 2022). Furthermore, they are also valuable resources for comparative genomics, facilitating the de novo assembly of closely related species using reference-guided approaches (Lischer and Shimizu 2017).

### Genomic Population Structure

Genome-wide and mitogenomic population structure analyses supported the existence of two populations between the ATL and IDWP oceans. In addition, the mitochondrial DNA (mtDNA) showed a much higher differentiation than genome-wide markers; the higher *F*_ST_ and Φ_ST_ values obtained by mtDNA are explained by the reduced *N_e_* of haploid genomes, which results in faster genetic drift and earlier lineage sorting (Gandra et al. 2021). Even though previous studies, based on traditional markers, revealed the existence of four populations along the sailfish's distribution: ATL, Arabian Gulf, IDWP, and Eastern Pacific (Hoolihan et al. 2004; Graves and McDowell 2015; Lu et al. 2015; Rubio-Castro et al. 2016; Ferrette et al. 2021).

Our results corroborate these previous results, implying a single population within the ATL Ocean and another in the IDWP Ocean that accounts for the Indian and Western and Central Pacific oceans. The reconnaissance of these populations as distinct fisheries management units contradicts the current strategies adopted for the sailfish by the International Commission for the Conservation of Atlantic Tunas (ICCAT), the Indian Ocean Tuna Commission (IOTC), and the Western and Central Pacific Fisheries Commission (WCPFC), which traditionally manage the sailfish as eastern and western stocks in the ATL and Pacific oceans and as a single stock in the Indian Ocean. So, to counteract the overfishing effects on its populations (Hilborn et al. 2020), a new management strategy should be implemented, considering both populations as fishery management units, and different RFMOs should join efforts to implement an integrated management plan in the IDWP.

Sailfish exhibited different migration patterns for genome-wide and mitogenomic markers. Genome-wide short-term migration rates showed that migration occurs within the ATL and IDWP basins, with almost no migration between the ATL and Indo-Pacific oceans. However, the mitogenomic long-term migration rates connected these regions through a westward stepping-stone migration pattern from the WCP toward the ATL Ocean. Despite being a highly migratory species, no transoceanic migration between the ATL and Indo-Pacific is known for the sailfish; its displacements are more restrictive than other billfishes, and it may present site fidelity, cyclic annual movements, or both (Ortiz et al. 2003; Lam et al. 2016; Madigan et al. 2021).

Understanding the migration patterns between populations and their correlation with oceanic physicochemical is crucial for guiding management practices in commercially exploited species (DiBattista et al. 2017; Diopere et al. 2018). Yet, many tropical species can tolerate a narrower environmental range (Barron 1995) like the sailfish that inhabits moderately warm and productive areas, while oxygen minimum zones and sea surface temperatures (SSTs) <23°C may impose a barrier to its migration (Prince et al. 2010; Reygondeau et al. 2012; Braun et al. 2015; Lam et al. 2016; Thoya et al. 2022); therefore, minor climate changes may trigger substantial biological responses.

### Seascape Genomics

Detecting local adaptation can be challenging for highly migratory marine species formed by large and interconnected populations (Grummer et al. 2019). Even so, our results found a significant correlation between the outlier loci and four environmental features: chlorophyll and salinity concentrations, sea surface temperature, and dissolved oxygen. Changes in these variables could hamper the sailfish's dispersal between these two ocean basins leading to an even higher population structure that may trigger allopatric speciation and, thus, reproductive isolation between the populations, which may imply that the sailfish lacks the physiological and phenotypic plasticity to adapt to future environmental changes and, therefore, climate change can threaten the sustainability of sailfish fisheries worldwide (Stramma et al. 2012; Booth et al. 2017; Schneider and Meyer 2017). Nevertheless, the most significant environmental variable to explain the differences between the sailfish populations was isolation by distance (IBD) (Wright 1943; Sexton et al. 2014) and not isolation by environment (IBE) (Wang and Bradburd 2014).

### Demographic History and Genomic Diversity

Nakamura (1980) proposed the existence of two species in the genus *Istiophorus* and the fossil-calibrated tree showed that the sailfish's mitogenomic lineages diverged around 1.81 Ma at the end of the Early Pleistocene (2.58–1.80 Ma). A few individuals from ATL clustered within the IDWP clade, as also evidenced by Ferrette et al. (2021). On the other hand, our SNP-based phylogenetic inference showed that each population forms a distinct and statistically supported clade. Moreover, our nuclear results show no admixture among these clades/populations. Nevertheless, given the low raw genetic distances and pairwise *F*_ST_ estimates (supplementary fig. S12 and S20, Supplementary Material online), these clades/populations cannot be considered distinct species (Roux et al. 2016). Therefore, the sailfish is a monospecific genus represented by *I. platypterus*. In addition, the fossil-calibrated phylogeny also showed that the billfishes diverged during the Middle Miocene (15.97–11.63 Ma) although the extant species diverged during the Pliocene (5.33–2.58 Ma), as proposed by Santini and Sorenson (2013). However, the billfishes’ radiation occurred during the Miocene (Gracia et al. 2022).

The Plio–Pleistocene transition (PPT) (∼3.3–2.8 Ma) paved the way for the interglacial cycles of the Pleistocene, resulting in cooler temperatures between the ATL and Indo-Pacific oceans (Peterson et al. 2020). In addition, the closure of major oceanic gateways, such as the Isthmus of Panamá and the Indonesian Throughflow constriction, are both accountable for the escalation of glacial conditions during the PPT strengthening phylogeographic barriers worldwide (Bahr et al. 2022). According to our results, the two deep evolutionary lineages diverged during this climate context at the end of the Early Pleistocene.

Although based on mtDNA-based analyses, some ATL individuals clustered with Indo-Pacific individuals, the nuclear genome analyses indicate that individuals from the two ocean basins cluster as distinct units, with no evidence for past or ongoing gene flow. This mito-nuclear discordance appears consistent with a scenario of incomplete lineage sorting (ILS), whereby the random loss and fixation of a single locus (like mtDNA) can result in gene trees that are discordant with the species tree (true population history). This scenario would entail that the ancestral mtDNA haplotypes, which now occur exclusively in the ATL population, occurred originally widespread but were lost from the Indo-Pacific Ocean, possibly due to population bottlenecks during the Pleistocene glaciations. This scenario would explain the paraphyletic clustering of mtDNA haplotypes and the deep split times of the major mtDNA haplotype cluster. If the loss of mitochondrial haplotype diversity in the Pacific results from a population bottleneck, it would also be consistent with the observed differences in genome-wide heterozygosity between the populations (ATL: 0.228% vs. Indo-Pacific: 0.203%) (supplementary figs. S23, Supplementary Material online).

Although many widely distributed taxa are in monotypic genera or families, a circumtropical distribution may reduce the chances of allopatric speciation, which relies on a sufficient period of geographical isolation (Rocha and Bowen 2008; Gaither et al. 2016). However, speciating and/or recently speciated allopatric taxa might evolve under similar rather than divergent selective pressures contradicting the classical understanding of divergent adaptation as a trigger of speciation (Anderson and Weir 2022). In this sense, allopatric speciation does not, in general, require lineages to exploit new habitats or adapt to different ecological pressures but relies instead on their period of isolation, which leaves open the possibility that ecological divergence is significant after allopatric speciation as lineages expand their distribution ranges (Rundell and Price 2009; Anderson and Weir 2021). This might suggest that if the sailfish populations could remain isolated for enough time, given a biogeographic barrier between the ATL and Indo-Pacific oceans, both lineages could evolve into distinct allopatric species and/or locally adapted to the environmental characteristics of each ocean basin. Nonetheless, migration routes between the ATL and Indian oceans were closed during glacial intervals. However, the seasonality between interglacial cycles may have proportionate periods of warm water corridors, which allowed secondary contact between these lineages, counteracting the allopatric speciation (Ferrette et al. 2021). So, there is not enough genetic evidence to support distinct species for the monotypic genus *Istiophorus*, which is currently represented only by *I. platypterus*, as suggested by Collette et al. (2006). Nevertheless, an integrative taxonomic review is required for the genus *Istiophorus* to understand the taxonomic status of these highly divergent lineages.

Both populations have similar demographic histories during the Late Pleistocene. ATL underwent a bottleneck event, due to the climatic changes over the Last Glacial Period (120–11.5 Kya), which led to a drop in global sea levels (Spratt and Lisiecki 2016; Lynch-Stieglitz 2017). In the last 50 Kya, a major global glaciation culminated in the Last Glacial Maximum (LGM; ∼26.5–19 Kya), which corresponds to the maximum volume of global ice sheets and lowest global sea levels up to 130 month (Clark et al. 2009; Hughes et al. 2013). During this period, there was an increase in glacial conditions in the Southern Hemisphere and a weakening in Agulhas Leakage, a warm and saline water flow from the Indo-Pacific toward the ATL Ocean through South Africa (Simon et al. 2013; Civel-Mazens et al. 2021).

This climatic phenomenon could have functioned as a phylogeographic barrier for individuals between the ATL and Indian oceans, isolating ATL individuals from the Indo-Pacific. On the other hand, the LGM does not seem to have negatively affected the demography of IDWP. Individuals from the WCP and SEI showed a continuous *N_e_* expansion trend, regardless of whether a land bridge separated Western Pacific and Eastern Indian oceans during the Late Pleistocene (Ludt and Rocha 2015).

The sailfish's populational average genome-wide heterozygosity is low (ATL: 0.228%, IDWP: 0.203%), even among billfishes; it was smaller than the swordfish (0.365%) and the blue marlin (0.262%), although higher than the white marlin (0.102%) and the shortbill spearfish (0.177%) (supplementary fig. S17, Supplementary Material online). Yet the heterozygosity is within the range of other fishes (0.10–1.76%) (Tigano et al. 2021; Barry et al. 2022). Genome-wide heterozygosity measures genetic diversity across the genome and can evidence the population's long-term potential for adaptation and resilience in an evolving environment. Therefore, it is crucial for conserving and managing wild populations and genetic resources (Schmeller et al. 2018; Gandra et al. 2021).

Low genetic diversity indicates inbreeding depression or increased genetic drift, resulting from a recent populational reduction or a long-term small *N_e_* (Peery et al. 2012; Palsbøll et al. 2013). In contrast, high genetic diversity levels can promote long-term population survival and guarantee adaptive potential under climate change scenarios (Markert et al. 2010).

Despite the past fluctuations in *N_e_* due to climatic changes, overfishing is currently the main threat to marine harvested populations and can reduce the *N_e_* leading to the loss of genetic variation even in abundant and large populations (Pinsky and Palumbi 2014; Di Minin et al. 2019; Wolf et al. 2022). Nowadays, there is an increasing concern over the harmful genetic effects of overfishing on marine fishes, as it could increase extinction risks and decrease the fisheries management unit's recovery rates (Allendorf et al. 2008; Palkovacs 2011). Furthermore, overfishing may trigger fisheries-induced evolution favoring faster life histories (e.g., earlier maturation, increased reproductive investment, and reduced post-maturation growth) because of the high and size-selective mortality resulting from the fishery activity (Heino et al. 2015; Hollins et al. 2018).

In addition, inbreeding can reduce fitness over generations, leading to an inbreeding depression by increasing homozygosity at recessive alleles or decreasing heterozygosity (Keller and Waller 2002; Charlesworth and Willis 2009). Even so, on average, the sailfish exhibited low *F*_RoH_, showing no inbreeding, given the lack of long fragments of RoHs, which proves that the inbreeding events must have happened many generations ago. Large and panmictic populations commonly present shorter RoHs, whereas isolated or bottlenecked populations have a prevalence of longer RoHs.

### Management and Conservation

Overfished stocks have increased by 24.2% since the late 1980s, and overfishing is currently the main threat to ocean biodiversity (FAO 2020; Yan et al. 2021). This is no different for the sailfish, fishery stocks in the ATL and Indian oceans are subject to overfishing with a decrease in abundance (Worm and Tittensor 2011; Punt et al. 2015; Sharma et al. 2018), and very recently the species was listed as “vulnerable” on the IUCN Red List (Collette et al. 2022a). However, given the uncertainties about its fishery stock assessments and the lack of continuous time series of genome-wide heterozygosity measurements, fishing activity's effects on its populations are unclear because fisheries-induced evolution would be relevant to fisheries management if it happens across time (Law 2007). Nevertheless, even marine species severely affected by overfishing for extended periods, such as the ATL cod (*Gadus morhua*), may sustain healthy levels of genetic diversity if management strategies were adopted to strengthen the efforts to cease overfishing and reduce fishing pressure within the maximum sustainable yield (Pinsky et al. 2021; Juan-Jordá et al. 2022). In order to promote straightforward and affordable genomic approaches for future fishery stock evaluations, we provided a subset of 200 highly informative SNPs that can be applied for future sailfish fishery stock assessments with the same accuracy as the whole data set from this study, democratizing access to these techniques.

Genetics, species, and ecosystem diversities are the main pillars of biodiversity (DeWoody et al. 2021), although international conservation policy agencies and decision-makers have continuously neglected their monitoring. Although the IUCN Red List recognizes that genetic exchange is an essential criterion for delimiting populations, it does not systematically incorporate genetic concepts or data into its assessments (Willoughby et al. 2015; Garner et al. 2020). Therefore, we propose that IUCN includes an additional criterion that addresses *N_e_* and genome-wide heterozygosity monitoring for use in ranking conservation and management priorities for the sailfish and other fishery resources.

To conclude, the new chromosome-level reference genome assembly will supply a powerful tool to improve sailfish management and conservation actions; coupled with our whole-genome resequencing data set and the subset of 200 highly informative SNPs, they will serve as a starting point for implementing a genomic monitoring plan. In this sense, sailfish fisheries management should incorporate time series estimations of genome-wide heterozygosity, inbreeding, *N_e_*, and genomic population structure. In addition, given its short generation time (4.3 years), it, along with the reduction in sequencing costs, will provide valuable and unprecedented information to detect oscillations in the genetic health of fishery stocks.

## Material and Methods

### Sampling and Whole-genome Sequencing

Muscle tissue samples were collected from 60 sailfishes by fishing fleets, including large-scale pelagic longlines, small-scale gillnets, and recreational fisheries from the ATL, Indian, and Pacific oceans. Besides, one individual blue marlin (*Makaira nigricans*), white marlin (*Kajikia albida*), and shortbill spearfish (*Tetrapturus angustirostris*) were collected from the ATL Ocean (fig. 1). These samples were preserved in absolute ethanol and stored at −80 °C. High molecular weight genomic DNA was extracted using a standard phenol/chloroform protocol. Whole-genome paired-end short-read sequencing libraries were prepared for all 60 samples of the sailfish and three more for the marlins and spearfish with the NEBNext® Ultra™ II DNA Library Prep Kit for an insert size of 350 bp (2 × 150 bp). Polymerase Chain Reaction (PCR) products were purified and size selected with Agencourt^®^ AMPure^®^ XP (Beckman Coulter^©^, Indianapolis, USA); size distribution was analyzed in Agilent 2100 Bioanalyzer (Agilent Technologies, USA) and quantified by real-time PCR to ensure a minimum concentration of 3 nM, and then sequenced in a NovaSeq 6000 (Illumina^©^, San Diego, USA). Short-read data of an previously published Indo-Pacific sailfish (SRA: SRS7572558) and swordfish (*Xiphias gladius*) (SRA: SRR12883662) (Wu et al. 2021) were added to the downstream analyses.

To assemble the chromosome-level genome, we prepared a PacBio SMRTbell™ long-read library following the instructions of the SMRTbell™ Express Prep kit v2.0 sequencing on the Sequel System II (Pacific BioSciences^©^, Menlo Park, CA, USA) in continuous long-read (CLR) mode. The quality of the final library was assessed on the TapeStation System (Agilent Technologies^©^) using the Genomic DNA ScreenTape Analysis kit and the Invitrogen™ Qubit™ Fluorometer using the Qubit™ dsDNA high sensitivity (HS) assay kit (Thermo Fisher Scientific, Waltham, MA, USA). The high-quality high-throughput chromosome conformation capture (Hi-C) reads (SRA: SRR12883658) from Wu et al. (2021) were employed for chromosome-scale scaffolding.

### De Novo Genome Assembly

A long-read–based de novo genome assembly was generated with wtdbg2 v2.5 (Ruan and Li 2020) under the default parameters for PacBio Sequel reads, followed by a two-step polishing approach. First, three iterations of long-read polishing with racon v1.4.20 (Vaser et al. 2017) were performed followed by three iterations of short-read polishing with *pilon* v1.24 (Walker et al. 2014) to correct for random errors and single-base errors using the high-accuracy short reads with 50× mean coverage. The polished contig assembly was scaffolded twice with SSPACE-LongReads v1-1 (Boetzer and Pirovano 2014) using the long reads, followed by three iterations of gap closing using TGS-GapCloser v1.0.3 (Xu et al. 2020) to fill sequencing gaps between contigs introduced by the first scaffolding. Furthermore, the published Hi-C reads from Wu et al. (2021) were used to anchor contigs and initial scaffolds into chromosome-scale scaffolds using Juicer v1.6 (Durand et al. 2016a) and 3D-DNA (Dudchenko et al. 2017). The resulting Hi-C contact map was then used to manually curate the assembly in Juicebox v2.15.08 (Durand et al. 2016b) before two additional iterations of gap closing.

Assembly statistics were computed by QUAST v5.0.2 (Gurevich et al. 2013), and a gene set completeness analysis was conducted with BUSCO v5.2.2 (Manni et al. 2021) with the database for Actinopterygii orthologous genes (actinopterygii_odb10) and the “*--long*” option for optimization. Qualimap v2.2.1 (Okonechnikov et al. 2016) estimated mapping rates and coverage distribution after mapping both the long and short reads onto the assembly using Minimap v2.17 (Li 2018) and BWA-MEM v0.7.17 (Li and Durbin 2009), respectively. The assembly's completeness and quality were estimated with a k-mer–based approach using Merqury v1.3. (Rhie et al. 2020). Finally, the synteny between the Indo-Pacific sailfish genome from Wu et al. (2021) (GenBank: GCA_016859345.1) and our newly de novo assembly from an ATL sailfish was compared using JupiterPlot v1.0 (Chu 2018).

The repeats’ annotation was done in three steps. First, we used RepeatMasker v4.1.2 (http://www.repeatmasker.org) to annotate and hard mask known Actinopterygii repeats from the RepBase library, which comprises a database of representative eukaryotic repetitive sequences (Bao et al. 2015). Second, a de novo library of repetitive regions and transposable elements was created from the hard-masked genome assembly using RepeatModeler v2.0.2a (Flynn et al. 2020). A summary of repetitive elements and the relative abundance of repeat classes in the genome are shown in supplementary table S1 and supplementary figure S1, Supplementary Material online.

### Processing of Whole-Genome Resequencing Data

Fastp v0.23.2 evaluated the short reads for adaptors contamination, applied a low complexity filter and base error correction, restricted the presence of “*N*” bases to 5 per read, allowed a maximum of 40% of unqualified bases (≤Q15), applied trimming in a sliding window of 4 bp, discarded reads with ≤Q15, and removed polyG tails (Chen et al. 2018). The filtered reads were mapped against our genome assembly with BWA-MEM v0.7.17, generating sorted and indexed BAM files with SAMtools v1.14 (Li et al. 2009). PCR and optical duplicates were marked with Picard v2.26.5 (http://broadinstitute.github.io/picard) using the recommended parameters for patterned flow cells. Qualimap v2.2.1 was used to evaluate mapping quality. Next, BAM files were processed to find intervals that needed realignment with RealignerTargetCreator and then realigned by IndelRealigner with Genome Analysis Toolkit (GATK) v3.8-1 (https://gatk.broadinstitute.org/). SAMtools was used to remove reads that did not pass the described filters above keeping only unique reads that mapped properly in the realigned BAM files. Reads mapped to scaffolds shorter than 1 Mb (∼2% of the total assembly length) and repetitive regions were also removed from the filtered BAM files.

Genotype likelihoods from variant sites were estimated by SAMtools-modified Maq model in ANGSD v0.935 (Korneliussen et al. 2014). Mapping qualities were adjusted for excessive mismatches; therefore, reads and nucleotides with base quality <30 were discarded. Minimum and maximum thresholds for the global site depth were set to *d* ± 5 × MAD, where *d* is the global site depth distribution median and MAD is the median absolute deviation. Sites with a *P* value < 1 × 10^−6^ for strand bias, heterozygous bias, or Hardy–Weinberg equilibrium were excluded. Only biallelic SNPs called with a *P* value < 1 × 10^−6^, minimum minor allele frequency (MAF) of 0.025, and shared for at least 90% of individuals were kept for further analyses.

Pairwise LD was estimated by *ngsLD* v1.1.1 (Fox et al. 2019), assuming a maximum distance of 200 Kbp between the SNPs. The thresholds for LD pruning were randomly sampling 0.05% of all SNP pairwise combinations to fit an LD decay model for *r*^2^ values of SNP pairs distant up to 100 Kbp assuming 250 of bin size. Linked sites were pruned considering a maximum distance of 25 Kbp between SNPs and a minimum *r*^2^ of 0.1 (supplementary fig. S2, Supplementary Material online).

### Genomic Population Structure

The Kinship-based INference (KING) (Manichaikul et al. 2010) evaluated the relatedness between individuals to remove those with a high kinship coefficient (>0.5). Thus, the sample SFA18 was removed for downstream populational analysis due to the high relatedness with sample SFA16 (supplementary fig. S3, Supplementary Material online). A DAPC was estimated from LD-pruned SNPs with the R package *adegenet* v2.1.7 (Jombart and Ahmed 2011). The number of principal components (PCs) kept for the discriminant analysis (DA) was chosen through a cross-validation test performed on a training set comprising 90% of the observations in each subpopulation and then used to predict the groups of 10% of the remaining observations. Thus, the number of PCs associated with the lowest mean squared error (MSE) was kept. Individuals’ admixture proportions were estimated by NGSadmix (Skotte et al. 2013), assuming *K* values from 1 to 6 with 100 replicates each. For higher accuracy, setting a tolerance of 1 × 10^−6^ for convergence and a value of 0.9 for considering a site as missing to include only high-quality genotypes. The Δ*K* method selected the best number of clusters (*K*) (Evanno et al. 2005). In order to create a subset of highly informative SNPs for population genomic essays aiming at sailfish fishery stocks delimitation, the 200 SNPs with the highest standard deviation of allele frequencies between the two populations (with the additional requirement that both the minor allele and major allele are present in both populations) were selected.

The contemporary migration patterns were estimated by BayesAss3-SNPs (Mussmann et al. 2019). BA3-SNPS-autotune (https://github.com/stevemussmann/BA3-SNPS-autotune) was used with default settings for 1 million generations with 10% burn-in to tune-mixing parameters by implementing a binary search algorithm and conducting short exploratory runs to choose optimized parameters of migration rates, allele frequencies, and inbreeding coefficients. BayesAss3-SNPs ran for three independent runs performed through 100 million generations with 10% of burn-in with mixing parameters of 0.55 for migration rates, 0.55 for allele frequencies, and 0.075 for inbreeding coefficients.

IQ-TREE v2.1.3 (Minh et al. 2020) estimated a ML phylogenetic tree from variable SNPs, using a full tree search for model selection to increase accuracy, optimized base frequencies by ML, and ascertainment bias correction (ASC). The tree was estimated with 100,000 ultrafast bootstraps approximation (UFBoot), optimizing each bootstrap tree using a hill-climbing nearest-neighbor interchange (NNI). The blue and white marlins and the shortbill spearfish were set as outgroups.

### Seascape Genomics

Three genome scans were employed by the R package SambaR v1.07 (De Jong, De Jong, et al. 2021) using OutFLANK v0.2 (Whitlock and Lotterhos 2015), *pcadapt* v4.3.3 (Privé et al. 2020), and GWDS (De Jong, Lovatt, et al. 2021) to identify outlier loci under balancing or diversifying selection. Marine environmental data layers for ecologically relevant variables (supplementary fig. S14, Supplementary Material online) were collected by Bio-ORACLE v2.2 (Assis et al. 2018) and standardized with the “*decostand*” function. Multicollinearity between the environmental variables was estimated by a square root of the variance inflation factor (VIF), followed by a forward selection on adjusted *R*^2^ and *P* values of variables to guarantee non-correlated variables and to choose the most significant explanatory variables (supplementary tables S4 and S5 and supplementary figs. S4 and S5, Supplementary Material online).

To model linear relationships among environment predictors and outliers’ genomic variation identifying allele frequencies associated with the multivariate environment, a constrained RDA was estimated by the R package *vegan* v2.6–2 (Oksanen et al. 2020). The adjusted *R*^2^, number of observations, and degrees of freedom in the fitted model were adjusted for the number of explanatory variables. The significance of the global RDA, axes, and explanatory variables were assessed with 10,000 permutations (supplementary tables S4–9, Supplementary Material online). Allele frequencies were estimated by *adegenet*. Distance-based Moran's eigenvector maps (dbMEM) were computed from a geographic distance matrix by *adespatial* v0.3–18 (Dray et al. 2012).

### Historical Demography, Heterozygosity, and Inbreeding

Long-term changes in effective population sizes (*N_e_*) over time were estimated with the PSMC model (Li and Durbin 2011) using consensus genome sequences generated by BCFtools v1.14 (Li 2011) for the sailfish, the blue and white marlins, the spearfish, and the swordfish. Sites with read depth below to a third of the average depth or above twice each sample's median depth and with a consensus base quality <30 were removed. PSMC was executed using 25 iterations with a maximum 2*N*_0_-scaled coalescent time of 15, an initial θ/ρ ratio of 5, and 64 atomic time intervals (4 + 25 × 2 + 4 + 6) to infer the scaled mutation rate, the recombination rate, and the free population size parameters, respectively. We performed 100 bootstrap replicates by randomly sampling with replacement 1-Mb blocks from the consensus sequence for all individuals.

In addition, a nonparametric method was implemented by Stairway Plot v2.1.1 (Liu and Fu 2020) using unfolded SNP frequency spectra to estimate the recent in *N_e_* changes over time. The site frequency spectrum (SFS) was estimated per population by ANGSD v0.935 and scaled by a substitution rate of 2.2 × 10^−9^ substitutions per site per generation estimated for the sailfish and 1.2 × 10^−9^ for the swordfish (Wu et al. 2021) and the appropriate generation length per species: sailfish (4.3), blue marlin (4.5), white marlin (5.5), shortbill spearfish (4.0), and swordfish (6.5 years) (Collette et al. 2022a, 2022b, 2022c, 2022d). Furthermore, we used GONE GitHub *commit ae98486* (Santiago et al. 2020) to infer the demographic history of a population within the past 100 generations from the observed spectrum of LD using the default parameters, except for the number of loci that was increased to 100.000 SNPs per chromosome, MAF = 0.025, and 100 replications.

BCFtools was used to estimate the genome-wide heterozygosity as the proportion of heterozygous sites. To evaluate the populational inbreeding level, genome-wide RoH were estimated with BCFtools through a hidden Markov model (HMM), estimating the allele frequency by recalculating INFO/AC and INFO/AN using the genotype likelihoods FORMAT/PL (“PL”). The proportion of the genome covered by RoH (*F*_RoH_) was used as a proxy for inbreeding (Narasimhan et al. 2016).

### Mitogenome Assembling, Annotation, and Population Analysis

Each mitogenome was assembled with GetOrganelle v1.7.4 (Jin et al. 2020) from whole-genome short reads using the default parameters to assembly animal mitogenomes, setting SPAdes kmer values of 21, 45, 65, 85, 105, and 10 maximum extending rounds and annotated by MitoAnnotator v3.70 (Iwasaki et al. 2013). We removed the 22 mitochondrial tRNAs, retaining the 13 protein-coding regions, 16S, 12S, and the control region comprising >90% of the mitogenomes length for downstream analyses. AMOVA was performed in Arlequin v3.5.2.2 (Excoffier and Lischer 2010). The posterior probability of population membership for individuals was inferred using a Bayesian model estimated by Geneland v4.9.2 (Guillot et al. 2005), performed under the spatial model assuming a correlated frequency model over ten independent Markov chain Monte Carlo (MCMC) simulations, varying *K* between 1 and 6 through 10 million MCMC iterations with a thinning of 1,000% and 10% burn-in. The best-supported *K* value was chosen based on the highest average log posterior probability score.

A Bayesian phylogenetic inference estimated the divergence time between sailfish lineages built by BEAST v2.6.6 (Bouckaert et al. 2014) setting an uncorrelated lognormal relaxed clock and a birth–death speciation model as priors. Fossil calibration followed Santini and Sorenson (2013) applying an exponential prior with an offset of 56 million years ago (Ma) and a mean of 10, leading to an age of 94 Ma in the Upper Cretaceous, the age of the oldest crown Acanthomorpha fossils for the soft upper bound. *Makaira* sp. fossil from the Middle Miocene was set as the minimum age for the crown Istiophoridae with an offset of 15 Ma and a mean of 14 as the soft upper bound. Posterior distributions were assessed by three independent runs of 500 million generations of MCMC with 10% burn-in. All billfish mitogenomes used for the phylogenetic inference are displayed in supplementary table S14, Supplementary Material online. We estimated extended Bayesian skyline plots (eBSP) (Heled and Drummond 2008) using a strict clock prior to infer variation of *N_e_* over time. The posterior distribution of parameters was estimated using three independent runs of 500 million generations of MCMC with 10% burn-in.

The number of historical migrants per generation (*Nm* = Θ × *M*) among populations was inferred by MIGRATE-N v4.4.4 (Beerli and Palczewski 2010), under a Bayesian approach. The historical mutation-scaled population sizes (Θ), defined as Θ = *N_e_*µ for mtDNA, and the historical mutation-scaled immigration rate (*M*), defined as *M* = *m*/µ, where *m* is the immigration rate and µ is the mutation rate per site per generation. The runs were performed with an exponential distribution range of Θ 0–0.1 (Δ 0.01; 5,000 bins) and *M* 0–1,000 (Δ 100; 5,000 bins). The MCMC settings were set as one long chain recorded by 300,000 steps incremented every 1,000 steps with a single replicate making up 300 million iterations and a static heating scheme of eight parallel chains with temperatures 1.0; 1.17; 1.4; 1.75; 2.33; 3.5; 7.0; and 1,000,000 with a burn-in of 30% per chain.

## Supplementary Material

evad042_Supplementary_Data

## Data Availability

The data underlying this article are available in GenBank assigned to the BioProject: PRJNA695102. BioSample: SAMN23026678–SAMN23026696, SAMN28005069–SAMN28005108, and SAMN30802609–SAMN30802611. SRA: SRR16903997–SRR16903979, SRR19018781–SRR19018742. GenBank Access Numbers: OP404092–OP404151, OP414774–OP414776. All scripts used in the downstream analysis are provided at https://github.com/bferrette/sailfish.
