# Supplementary material for: Seascape Genomics and Phylogeography of the Sailfish (Istiophorus platypterus)
Source: Genome Biol Evol. 2023 Mar 13;15(4):evad042. doi: 10.1093/gbe/evad042 (PMC10079183; doi:10.1093/gbe/evad042)
Supplement: evad042_Supplementary_Data [file evad042_supplementary_data.zip › Supplementary_Material_R1.pdf]

Seascape genomics and mitogenomic phylogeography of the sailfish (*Istiophorus platypterus*)

Bruno Lopes da Silva Ferrette[1,2,3], Raphael T. F. Coimbra[1,4], Sven Winter[1,5], Menno J. De Jong[1], Samuel Mackey Williams[6], Rui Coelho[7,8], Daniela Rosa[7,8], Matheus Marcos Rotundo[9], Freddy Arocha[10], Bruno Leite Mourato[11], Fernando Fernandes Mendonça[12], Axel Janke[1,2,4]

[1] Senckenberg Biodiversity and Climate Research Centre, Senckenberganlage 25, 60325, Frankfurt am Main, Germany

[2] LOEWE Centre for Translational Biodiversity Genomics, Senckenberganlage 25, 60325, Frankfurt am Main, Germany

[3] Conservation Genetics Laboratory, Santa Cecília University, Cesário Mota St., 8, 11045-040, Santos, Brazil

[4] Institute for Ecology, Evolution and Diversity, Goethe University, Max von Laue Strasse 13, 60438, Frankfurt am Main, Germany

[5] Research Institute of Wildlife Ecology, Vetmeduni Vienna, Savoyenstraße 1, 1160, Vienna, Austria

[6] School of Biological Sciences, The University of Queensland, St Lucia, 4072, Queensland, Australia

[7] Instituto Português do Mar e da Atmosfera (IPMA), Av. 5 de Outubro s/n°, 8700-305, Olhão, Portugal

[8] Centro de Ciências do Mar (CCMAR), Universidade do Algarve, Campus de Gambelas, 8005-139, Faro, Portugal

[9] Acervo Zoológico (AZUSC), Universidade Santa Cecília (UNISANTA), Oswaldo Cruz St., 258, 11045-907, Santos, Brazil

10 Instituto Oceanográfico de Venezuela, Universidad de Oriente (UDO), Camaná, Venezuela

[11] Laboratório de Ciências da Pesca (LABPESCA), Instituto do Mar (IMar), Universidade Federal de São Paulo (UNIFESP), Campus Baixada Santista, 144, Dr. Carvalho de Mendonça St., 11070-100, Santos, Brazil

[12] Laboratório de Genética Pesqueira e Conservação (GenPesC), Instituto do Mar (IMar), Universidade Federal de São Paulo (UNIFESP), Campus Baixada Santista, 144, Dr. Carvalho de Mendonça St., 11070-100, Santos, Brazil

*Author for Correspondence: bruno.ferrette@senckenberg.de

Table S1. Quality Assessment Tool for the sailfish genome assembly. Contigs is the total number of contigs of length ≥ x bp. Total length (TL) is the total number of base pairs (bp). The largest contig is the length of the longest contig in the assembly. Total length is the total number of bases in the assembly. GC (%) is the total number of G and C nucleotides in the assembly, divided by the total length. N50 is the length for which the collection of all contigs of that length or longer covers at least half an assembly. N75 is similar to N50 but with 75%. L50 and L75 are the number of contigs equal to or longer than N50 or N75. Ns per 100 Kbp is the average number of uncalled bases (Ns) per 100,000 assembly bases.

**Assembly**	**our study scaffolds**	**our study contigs**	**Wu *et al.* (2021) scaffolds**	**Wu *et al.* (2021) contigs**
**contigs ≥ 1000 bp**	402	444	471	635
**contigs ≥ 10,000 bp**	189	217	246	408
**contigs ≥ 100,000 bp**	28	47	32	126
**contigs ≥ 1,000,000 bp**	24	38	24	83
**contigs ≥ 10,000,000 bp**	24	26	24	18
**TL ≥ 1000 bp**	619,036,510	618,988,655	614,147,335	614,064,021
**TL ≥ 10,000 bp**	617,689,266	617,579,414	612,989,320	612,895,040
**TL ≥ 100,000 bp**	614,513,932	614,125,598	607,029,199	605,055,845
**TL ≥ 1,000,000 bp**	612,904,761	610,902,534	605,656,581	586,243,237
**TL ≥ 10,000,000 bp**	612,904,761	573,710,942	605,656,581	321,590,000
**contigs**	404	447	471	636
**Largest contig**	32,784,910	31,242,566	33,078,667	33,078,667
**Total Length**	619,037,273	618,990,261	614,147,335	614,064,835
**GC (%)**	41	41	41	41
**N50**	26,279,568	23,352,486	26,283,175	11,851,605
**N75**	23,793,836	19,051,225	23,577,423	5,656,603
**L50**	11	12	11	17
**L75**	17	19	17	37
**Ns per 100 Kbp**	7.59	0	13.43	0

Table S2. Repeat content of the genome assembly. **Class**, class of repetitive regions. **Count**, number of times the repetitive region is present. **bpMasked**, number of base pairs masked; **%Masked**, percentage of base pairs masked. LINE, Long Interspersed Nuclear Elements (include retroposons); LTR, Long Terminal Repeat elements (include retroposons); SINE, Short Interspersed Nuclear Elements; RC, Rolling Circle.

Class	Count	bpMasked	%Masked
**ARTEFACT**	3	185	0.00%
**DNA**	245,310	38,735,424	6.24%
**LINE**	46,997	13,463,182	2.18%
**LTR**	26,030	3,827,551	0.62%
**RC**	8,150	1,027,304	0.17%
**Retroposon**	11	834	0.00%
**SINE**	10,214	1,156,446	0.17%
**Unknown**	243,753	40,759,942	6.58%
**Low complexity**	52,723	2,924,157	0.47%
**Satellite**	1,264	212,877	0.03%
**Simple repeat**	419,743	19,465,602	3.14%
**rRNA**	160	40,598	0.01%
**scRNA**	1	103	0.00%
**snRNA**	90	11,066	0.00%
**tRNA**	1,116	76,969	0.01%
**Total**	1,055,565	1.22E+08	19.66%

Table S3. Short reads mapping statistics. **Mapped Reads**, total reads in the bam file (millions); **%GC**, mean GC content; **Ins. size**, median insert size; **≥10X**, fraction of genome with at least 10X coverage; **Mean cov**, mean coverage; **% Align**, % mapped reads; **Error rate**, Alignment error rate total edit distance (SAM NM field) over the number of mapped bases.

Sample Name	Mapped Reads	%GC	Ins. size	≥10X	Mean cov	%Aligned	Error rate
**BUM01**	233	43%	350	97.50%	39.1X	98.60%	2.29%
**SFA01**	82.3	43%	340	73.10%	13.3X	99.30%	0.69%
**SFA02**	84.5	41%	341	79.10%	13.9X	98.20%	0.68%
**SFA03**	89.4	41%	348	94.80%	18.7X	98.10%	0.67%
**SFA04**	89.6	41%	347	94.40%	18.3X	93.30%	0.66%
**SFA05**	81.7	42%	363	82.30%	14.0X	96.60%	0.70%
**SFA06**	78.7	42%	334	82.10%	14.0X	98.30%	0.67%
**SFA07**	85.3	42%	348	85.60%	14.7X	99.00%	0.68%
**SFA08**	80.5	42%	344	82.50%	14.1X	97.20%	0.67%
**SFA09**	81.7	42%	345	84.00%	14.4X	98.90%	0.67%
**SFA10**	82.7	42%	352	81.70%	13.9X	97.40%	0.68%
**SFA11**	80.8	42%	361	79.30%	13.5X	97.50%	0.72%
**SFA12**	79.6	42%	297	84.30%	14.6X	98.80%	0.67%
**SFA13**	78.4	42%	354	76.30%	13.0X	98.30%	0.70%
**SFA14**	83.3	42%	302	81.30%	14.3X	97.10%	0.73%
**SFA15**	103.2	41%	353	96.60%	20.9X	98.80%	0.66%
**SFA16**	115.4	41%	344	97.30%	23.0X	99.20%	0.67%
**SFA17**	90.2	41%	352	94.70%	18.5X	99.10%	0.67%
**SFA18**	101	41%	359	96.30%	20.3X	97.90%	0.69%
**SFA19**	119.2	41%	358	97.50%	23.7X	98.90%	0.68%
**SFA20**	84.2	41%	349	92.90%	17.3X	99.00%	0.67%
**SFA21**	95.5	42%	342	95.10%	19.3X	99.30%	0.66%
**SFA22**	97.5	42%	343	93.80%	17.9X	99.00%	0.72%
**SFA23**	97.9	42%	330	93.40%	17.9X	99.20%	0.70%
**SFA24**	84.6	41%	363	93.50%	17.5X	99.20%	0.66%
**SFA25**	102.3	41%	359	96.80%	20.9X	99.20%	0.67%
**SFA26**	95.5	41%	358	95.70%	19.4X	99.30%	0.67%
**SFA27**	101.6	41%	361	96.60%	20.9X	99.20%	0.67%
**SFA28**	89.9	41%	360	94.80%	18.4X	99.30%	0.67%
**SFA29**	204.4	45%	355	82.70%	37.4X	99.20%	0.48%
**SFA30**	83.3	41%	354	93.10%	17.1X	99.20%	0.68%
**SFA31**	88.3	42%	344	94.20%	18.2X	93.50%	0.70%
**SFA32**	81.3	42%	371	78.80%	13.3X	99.10%	0.68%
**SFA33**	83.3	41%	339	84.60%	14.7X	99.30%	0.68%
**SFA34**	82.9	42%	342	82.40%	14.1X	99.10%	0.67%
**SFA35**	99.4	43%	320	92.80%	18.2X	98.80%	0.71%
**SFA36**	94.6	42%	324	90.00%	16.6X	96.80%	0.68%
**SFA37**	96.9	42%	322	91.80%	17.2X	98.60%	0.70%

**SFA38**	96.1	42%	315	92.50%	17.5X	99.00%	0.69%
**SFA39**	103.1	43%	317	92.50%	18.3X	98.90%	0.68%
**SFA40**	92.5	42%	323	91.50%	17.1X	99.00%	0.68%
**SFA41**	100.8	42%	314	94.20%	18.7X	98.60%	0.69%
**SFA42**	99.1	42%	306	93.10%	18.2X	99.10%	0.68%
**SFA43**	95.1	42%	305	92.40%	17.6X	98.40%	0.69%
**SFA44**	104	42%	335	93.20%	18.0X	96.80%	0.71%
**SFA45**	80.5	43%	332	74.60%	13.9X	99.30%	0.70%
**SFA46**	95	44%	323	89.90%	18.9X	96.00%	0.75%
**SFA47**	79.7	42%	259	78.00%	14.1X	98.70%	0.64%
**SFA48**	81.9	42%	271	81.50%	14.8X	99.20%	0.64%
**SFA49**	79.3	42%	297	80.50%	14.1X	98.90%	0.67%
**SFA50**	82.5	42%	301	84.90%	15.0X	99.00%	0.67%
**SFA51**	82.2	42%	330	80.30%	14.1X	99.40%	0.70%
**SFA52**	81.5	42%	357	84.70%	14.6X	99.40%	0.68%
**SFA53**	83.3	43%	379	77.90%	13.8X	99.40%	0.70%
**SFA54**	82.1	42%	370	84.00%	14.6X	99.20%	0.69%
**SFA55**	81.4	42%	329	80.60%	14.0X	99.40%	0.72%
**SFA56**	85.5	42%	339	85.50%	15.0X	99.40%	0.68%
**SFA57**	81.7	42%	365	80.70%	13.8X	99.20%	0.69%
**SFA58**	82.5	42%	354	78.70%	13.6X	99.40%	0.73%
**SFA59**	92.7	42%	355	95.20%	19.2X	99.40%	0.66%
**SFA60**	103.3	42%	353	96.40%	20.8X	99.10%	0.67%
**SFA61**	521.6	41%	331	99.10%	107.2X	99.00%	0.72%
**SPF01**	248.5	42%	329	96.70%	42.9X	98.90%	2.88%
**WHM01**	254.7	42%	323	97.70%	49.8X	98.90%	2.23%

Table S4. Square root of Variance Inflation Factor (VIF) multicollinearity test, where √VIF > 2 is considered high. bathy, mean bathymetry; chlo, mean concentration of surface chlorophyll; scvl, mean velocity of surface marine currents; sdO2, mean concentration of surface dissolved molecular oxygen; pH, pH estimations; ptpk, mean concentration of surface phytoplankton; sprp, daily mean of primary surface productivity; ssal, mean concentration of surface salinity; sst, mean sea surface temperature.

					**All**					
**bathy**	**chlo**	**scvl**	**sdO2**	**ph**	**ptpk**	**sprp**	**ssal**	**light**	**diff.atn**	**sst**
1.714	16.468	1.884	4.023	1.544	15.05	4.479	1.453	1.446	2.895	4.081
					**RDA1**					
**ssal**	**sst**	**chlo**								
1.169	1.215	1.061	-	-	-	-	-	-	-	-
					**RDA2**					
**chlo**	**sdO2**	**ssal**								
1.179	1.208	1.046	-	-	-	-	-	-	-	-

Table S5. Forward model selection for environmental variables for redundancy analysis on adjusted R^2 and *p*-values. ssal, mean concentration of surface salinity; sst, mean sea surface temperature; sdO2, mean concentration of surface dissolved molecular oxygen; chlo, mean concentration of surface chlorophyll.

variable	R^2_{adj}	Df	AIC	F Pr(>F)	*p*-value
**ssal**	0.148	1	237.07	11.271	4e-04 ***
**sst**	0.289	1	227.17	12.497	2e-04 ***
**chlo**	0.460	1	211.62	19.031	1e-04 ***
**sdO2**	0.527	1	204.58	8.942	2e-04 ***
**All**	0.531	-	-	-	-

*** $p \leq 0.01$.

Table S6. Global RDA1 significance.

	Df	Variance	F Pr(>F)	*p*-value
**Model**	4	61.092	40.176	1e-04 ***
**Residual**	55	20.908		

*** $p \leq 0.01$.

Table S7. Axis significance in RDA1.

	Df	Variance	F Pr(>F)	*p*-value
**RDA1**	1	60.075	158.028	0.0001 ***
**RDA2**	1	0.472	1.2427	0.676
**RDA3**	1	0.366	0.9641	0.824
**RDA4**	1	0.178	0.4683	0.995
**Residual**	55	20.908		

*** $p \leq 0.01$.

Table S8. Explanatory variables significance in RDA1. ssal, mean concentration of surface salinity; sst, mean sea surface temperature; chlo, mean concentration of surface chlorophyll, MEM1, Moran's Eigenvector Maps.

	Df	Variance	F Pr(>F)	*p*-value
**chlo**	1	11.171	29.387	1e-04 ***
**ssal**	1	9.542	25.101	1e-04 ***
**sst**	1	19.219	50.556	1e-04 ***
**MEM1**	1	21.158	55.658	1e-04 ***
**Residual**	55	20.908		

*** $p \leq 0.01$.

Table S9. Global RDA2 significance.

	Df	Variance	F Pr(>F)	*p*-value
**Model**	4	60.989	39.911	1e-04 ***
**Residual**	55	21.011		

*** $p \leq 0.01$.

Table S10. Axis significance in RDA2.

	Df	Variance	F Pr(>F)	*p*-value
**RDA1**	1	59.843	156.6455	0.0001 ***
**RDA2**	1	0.481	1.2583	0.672
**RDA3**	1	0.366	0.9582	0.831
**RDA4**	1	0.299	0.7824	0.759
**Residual**	55	21.011		

*** $p \leq 0.01$.

Table S11. Explanatory variables significance in RDA2. ssal, mean concentration of surface salinity; sdO2, mean concentration of surface dissolved molecular oxygen; chlo, mean concentration of surface chlorophyll, MEM1, Moran's Eigenvector Maps.

	Df	Variance	F Pr(>F)	*p*-value
**chlo**	1	11.172	29.243	1e-04 ***
**sdO2**	1	17.544	45.924	1e-04 ***
**ssal**	1	16.865	44.147	1e-04 ***
**MEM1**	1	15.407	40.33	1e-04 ***
**Residual**	55	21.012		

*** $p \leq 0.01$.

Table S12. Genome-wide heterozygosity and runs of homozygosity (RoHs) statistics per individual. **n_{RefHom}**, number of reference homozygous genotypes; **$n_{NonRefHom}$**, number of non-reference homozygous genotypes; **n_{Hets}**, number of heterozygous genotypes; **meanHet**, mean heterozygosity; **nTi**, number of transitions; **nTv**, num of transversions; **n_{Indels}**, number indels; **$n_{Singletons}$**, number of singletons; **n_{miss}**, number of missing genotypes; **F_{miss}**, frequency of missing genotypes; **n_{RoH}**, number of fragments of runs of homozygosity; **S_{RoH}**, sum of RoH fragments; **F_{RoH}**, frequency of RoHs. WCA, Western Central Atlantic; SWA, Southwest Atlantic; ECA, Eastern Central Atlantic; SWI, Southwest Indian; SEI, Southeast Indian; WCP, Western Central Pacific.

Sample	n_{RefHom}	$n_{NonRefHom}$	n_{Hets}	meanHet	nTi	nTv	n_{Indels}	$n_{Singletons}$	n_{miss}	F_{miss}	n_{RoH}	S_{RoH}	F_{RoH}
**SFA01**	475,406,702	536,349	1,068,428	0.00224	882,482	722,295	379,471	50,801	324,473	0.06798	968	15,917,380	0.02571
**SFA02**	475,413,620	532,921	1,083,722	0.00227	888,525	728,118	381,830	52,272	303,330	0.06355	978	15,765,253	0.02547
**SFA03**	475,571,024	531,574	1,099,580	0.0023	896,574	734,580	385,146	58,461	128,099	0.02684	1,114	16,478,820	0.02662
**SFA04**	475,564,989	532,391	1,100,560	0.00231	898,529	734,422	386,801	58,758	130,682	0.02738	1,082	15,081,816	0.02436
**SFA05**	475,496,364	533,731	1,080,808	0.00227	887,493	727,046	382,188	52,257	222,332	0.04658	1,106	18,149,237	0.02932
**SFA06**	475,507,514	531,776	1,082,925	0.00227	887,093	727,608	382,150	53,892	211,058	0.04422	1,065	16,471,913	0.02661
**SFA07**	475,519,465	534,611	1,087,665	0.00228	892,042	730,234	382,920	54,737	190,762	0.03996	1,098	18,802,357	0.03037
**SFA08**	475,499,183	532,690	1,082,351	0.00227	887,649	727,392	381,680	52,937	219,519	0.04599	1,059	17,743,242	0.02866
**SFA09**	475,504,402	536,484	1,080,554	0.00226	889,386	727,652	382,696	54,320	211,287	0.04426	1,153	21,081,291	0.03405
**SFA10**	475,512,718	529,244	1,087,301	0.00228	888,189	728,356	382,131	53,556	204,029	0.04274	1,007	15,912,615	0.02571
**SFA11**	475,497,033	534,171	1,077,266	0.00226	885,555	725,882	381,372	52,075	225,581	0.04726	1,035	16,368,167	0.02644
**SFA12**	475,474,038	534,180	1,084,269	0.00227	889,042	729,407	376,629	55,006	246,307	0.0516	1,093	16,569,151	0.02677
**SFA13**	475,491,731	530,762	1,067,588	0.00224	878,672	719,678	380,873	50,115	244,469	0.05122	1,072	17,734,515	0.02865
**SFA14**	475,483,509	517,034	1,112,394	0.00233	897,110	732,318	384,425	50,902	218,061	0.04568	816	14,026,400	0.02266
**SFA15**	475,581,884	536,361	1,098,397	0.0023	897,757	737,001	386,492	60,834	112,289	0.02352	1,150	16,982,717	0.02743
**SFA16**	475,581,149	536,373	1,106,288	0.00232	901,991	740,670	388,691	560	102,922	0.02156	1,009	14,622,462	0.02362
**SFA17**	475,564,896	539,818	1,087,890	0.00228	893,918	733,790	385,546	58,565	137,273	0.02876	1,072	19,895,984	0.03214
**SFA18**	475,569,294	535,875	1,104,364	0.00231	900,792	739,447	390,963	388	114,927	0.02408	1,001	14,671,031	0.0237
**SFA19**	475,589,149	537,548	1,096,820	0.0023	897,930	736,438	383,451	61,267	108,455	0.02272	1,204	16,640,757	0.02688
**SFA20**	475,552,843	533,738	1,100,806	0.00231	897,482	737,062	386,696	57,854	141,340	0.02961	1,035	16,769,298	0.02709
**SFA21**	475,568,133	533,179	1,097,927	0.0023	896,804	734,302	385,531	59,087	130,653	0.02737	1,105	15,563,413	0.02514
**SFA22**	475,564,901	527,578	1,095,097	0.00229	891,648	731,027	385,027	55,167	142,820	0.02992	1,045	16,627,765	0.02686
**SFA23**	475,568,303	529,071	1,093,369	0.00229	892,009	730,431	384,421	56,028	140,259	0.02938	1,087	16,873,549	0.02726
**SFA24**	475,567,657	530,571	1,088,880	0.00228	890,103	729,348	385,711	56,461	142,604	0.02988	1,106	15,596,762	0.0252

**SFA25**	475,592,930	529,668	1,095,888	0.0023	892,019	733,537	384,889	59,139	112,048	0.02347	1,116	16,556,685	0.02675
**SFA26**	475,590,710	526,007	1,104,125	0.00231	895,629	734,503	386,977	58,109	107,604	0.02254	917	13,790,568	0.02228
**SFA27**	475,599,860	532,611	1,089,382	0.00228	891,327	730,666	385,005	56,690	108,565	0.02274	1,147	17,262,735	0.02789
**SFA28**	475,566,350	528,293	1,095,112	0.00229	891,085	732,320	386,284	55,911	139,384	0.0292	1,078	15,900,801	0.02569
**SFA29**	469,001,883	14,712	1,015,950	0.00216	570,377	460,285	278,140	25,399	7,404,738	1.55093	1,040	17,679,522	0.02856
**SFA30**	475,571,573	531,579	1,088,854	0.00228	890,844	729,589	384,457	56,656	138,960	0.02911	1,065	16,115,532	0.02603
**SFA31**	475,585,143	530,207	1,088,744	0.00228	890,617	728,334	385,194	57,131	126,135	0.02643	1,134	17,079,587	0.02759
**SFA32**	475,491,448	531,658	1,068,164	0.00224	879,372	720,450	380,115	50,566	244,038	0.05113	1,012	19,388,881	0.03132
**SFA33**	475,575,637	587,596	959,725	0.00201	849,538	697,783	381,080	51,880	211,385	0.04428	645	12,174,398	0.01967
**SFA34**	475,583,589	587,206	953,857	0.002	846,232	694,831	379,417	50,469	211,354	0.04428	645	12,880,780	0.02081
**SFA35**	475,640,033	585,470	970,148	0.00203	854,028	701,590	381,860	53,835	137,912	0.02889	680	9,875,287	0.01595
**SFA36**	475,625,968	584,360	969,667	0.00203	853,181	700,846	382,236	52,887	153,192	0.03209	587	9,179,956	0.01483
**SFA37**	475,629,106	587,027	964,627	0.00202	852,097	699,557	382,038	53,716	152,625	0.03197	679	12,092,635	0.01953
**SFA38**	475,631,584	585,673	968,468	0.00203	853,417	700,724	382,343	54,144	147,355	0.03087	620	10,429,391	0.01685
**SFA39**	475,642,024	585,774	967,379	0.00203	853,205	699,948	380,740	53,973	139,506	0.02923	646	10,388,061	0.01678
**SFA40**	475,633,691	586,109	970,155	0.00203	854,246	702,018	382,386	54,156	143,082	0.02998	618	9,325,642	0.01506
**SFA41**	475,654,516	583,911	971,680	0.00204	854,690	700,901	381,464	55,261	123,852	0.02595	620	9,780,452	0.0158
**SFA42**	475,647,584	584,473	967,528	0.00203	851,614	700,387	381,743	54,460	134,095	0.02809	644	10,714,712	0.01731
**SFA43**	475,646,196	583,919	971,152	0.00204	853,819	701,252	382,298	53,066	131,858	0.02762	588	8,944,633	0.01445
**SFA44**	475,647,763	583,614	970,563	0.00203	853,053	701,124	381,908	54,247	131,575	0.02756	586	9,874,094	0.01595
**SFA45**	475,509,185	583,659	953,687	0.002	845,399	691,947	378,145	48,703	290,747	0.06091	545	10,363,609	0.01674
**SFA46**	475,601,616	522,454	1,105,311	0.00232	893,879	733,886	389,941	52,228	96,101	0.02013	59	1,006,183	0.00163
**SFA47**	475,462,650	583,585	958,654	0.00201	846,979	695,260	371,802	48,622	338,732	0.07096	593	10,649,559	0.0172
**SFA48**	475,485,431	584,175	961,110	0.00201	848,909	696,376	373,239	50,798	311,468	0.06525	593	9,812,534	0.01585
**SFA49**	475,527,235	572,351	983,311	0.00206	854,104	701,558	376,018	50,102	256,508	0.05374	466	7,858,495	0.01269
**SFA50**	475,573,969	584,456	965,289	0.00202	851,268	698,477	374,906	52,055	216,803	0.04542	579	8,900,172	0.01438
**SFA51**	475,567,767	588,363	957,520	0.00201	849,532	696,351	379,823	48,498	221,950	0.0465	684	12,305,125	0.01988
**SFA52**	475,597,897	582,646	967,394	0.00203	851,019	699,021	380,043	51,791	187,443	0.03927	601	9,569,188	0.01546
**SFA53**	475,518,446	587,304	951,681	0.00199	845,895	693,090	378,748	49,465	279,244	0.0585	668	10,852,820	0.01753

**SFA54**	475,586,126	585,452	964,484	0.00202	851,772	698,164	381,437	50,273	197,924	0.04146	594	9,403,760	0.01519
**SFA55**	475,522,726	586,668	950,010	0.00199	843,672	693,006	378,125	48,432	277,894	0.05822	643	11,333,305	0.01831
**SFA56**	475,608,261	585,255	961,726	0.00202	849,838	697,143	381,024	51,788	179,157	0.03753	653	10,409,085	0.01681
**SFA57**	475,574,318	585,663	958,183	0.00201	848,082	695,764	380,487	49,803	216,772	0.04541	641	10,756,440	0.01738
**SFA58**	475,565,655	585,523	955,479	0.002	846,006	694,996	379,875	47,896	228,891	0.04795	592	10,661,443	0.01722
**SFA59**	475,652,915	585,989	970,174	0.00203	853,809	702,354	382,605	53,193	123,740	0.02592	654	10,003,423	0.01616
**SFA60**	475,668,116	585,037	973,913	0.00204	855,222	703,728	381,860	58,646	106,497	0.02231	682	9,983,656	0.01613
**SHS-SA01**	475,657,540	588,320	970,491	0.00203	855,619	703,192	381,770	57,819	117,302	0.02457	665	10,415,440	0.01683
**Average**	475,457,179	547,784	1,031,555	0.00216	867,708	711,631	380,545	51,608	298,360	0.06250	843	13,509,254	0.02182

Table S13. Analysis of Molecular Variance (AMOVA) with hierarchical genetic structuring scenarios among sampled regions. Fixation indexes defined as $\mathbf{\Phi_{ST}}$, the permutation of haplotypes among populations among groups; $\mathbf{\Phi_{SC}}$, the permutation of haplotypes among populations within groups; $\mathbf{\Phi_{CT}}$, the permutation of populations among groups; **%**, percentage of variation; ***p*-value**, the marginal significance within a statistical hypothesis test representing the probability of the occurrence of a given event. *, significant *p*-values after the Bonferroni correction ($p < 0.005$). WCA, Western Central Atlantic; SWA, Southwest Atlantic; ECA, Eastern Central Atlantic; SWI, Southwest Indian; SEI, Southeast Indian; WCP, Western Central Pacific.

Scenario	Hypothetical structuring scenarios	Source of Variation	%	Fixation indexes	*p*-value
**I**	**(WCA+SWA+ECA+SWI+SEI+WCP)**	Among populations	45.64	$\mathbf{\Phi_{ST} = 0.456}$	*
		Whitin populations	54.36		
**II**	**(WCA+SWA+ECA) X (SWI+SEI+WCP)**	Among groups	48.66	$\mathbf{\Phi_{CT} = 0.486}$	*
		Among populations within groups	7.15	$\Phi_{SC} = 0.139$	0.006
		Among populations among groups	44.2	$\mathbf{\Phi_{ST} = 0.558}$	*
**III**	**(WCA+SWA+ECA) X (SWI+SEI) X (WCP)**	Among groups	37.71	$\Phi_{CT} = 0.377$	0.135
		Among populations within groups	13.04	$\Phi_{SC} = 0.209$	0.004
		Among populations among groups	49.25	$\mathbf{\Phi_{ST} = 0.507}$	*
**IV**	**(WCA+SWA) X (ECA) X (SWI+SEI) X (WCP)**	Among groups	43.36	$\Phi_{CT} = 0.433$	0.091
		Among populations within groups	4.49	$\Phi_{SC} = 0.079$	0.033
		Among populations among groups	52.16	$\mathbf{\Phi_{ST} = 0.478}$	*
**V**	**(WCA+SWA) X (ECA) X (SWI) X (SEI) X (WCP)**	Among groups	44.6	$\Phi_{CT} = 0.446$	0.06614
		Among populations within groups	2.93	$\Phi_{SC} = 0.052$	0.147
		Among populations among groups	52.47	$\mathbf{\Phi_{ST} = 0.475}$	*

Table S14. Billfish mitogenomes from GenBank used in the construction of the fossil-calibrated Bayesian phylogenetic tree.

Common name	Species	GenBank Access Number
**black marlin**	*Istiompax indica*	NC_012675.1
**black marlin**	*Istiompax indica*	KJ510416
**black marlin**	*Istiompax indica*	KJ510417
**sailfish**	*Istiophorus platypterus*	NC_012676.1
**sailfish**	*Istiophorus platypterus*	KU315124
**white marlin**	*Kajikia albida*	NC_030010.1
**striped marlin**	*Kajikia audax*	NC_012678.1
**striped marlin**	*Kajikia audax*	KU315126
**blue marlin**	*Makaira mazara*	NC_012680.1
**blue marlin**	*Makaira nigricans*	NC_030006.1
**shortbill spearfish**	*Tetrapturus angustirostris*	NC_012679.1
**shortbill spearfish**	*Tetrapturus angustirostris*	KU315125
**Mediterranean shortbill spearfish**	*Tetrapturus belone*	NC_030008.1
**roundscale spearfish**	*Tetrapturus georgii*	NC_030009.1
**roundscale spearfish**	*Tetrapturus pfluegeri*	NC_030007.1
**swordfish**	*Xiphias gladius*	NC_012677.1

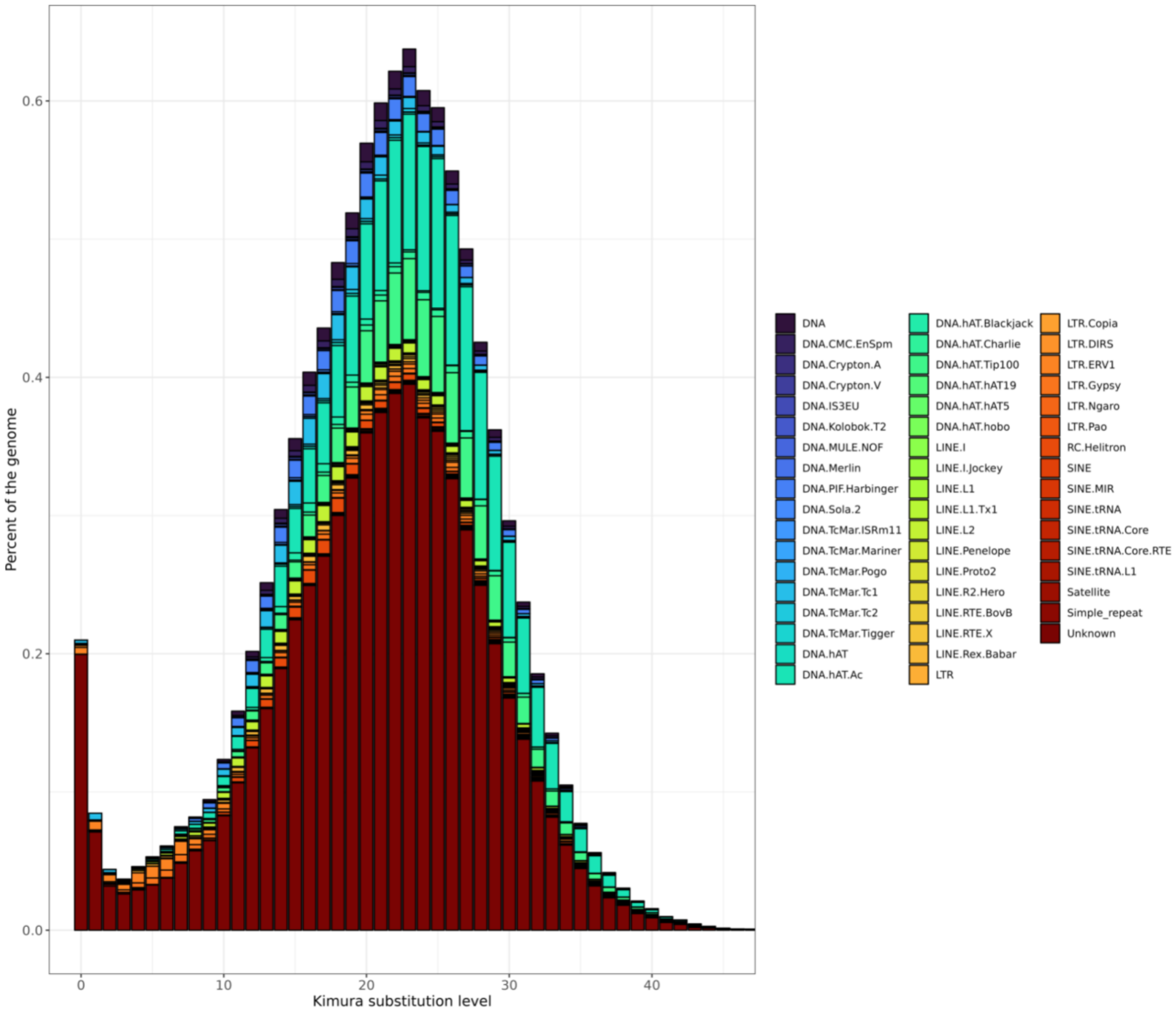

Fig S1. Repeat landscape. The repeat landscapes depict the relative abundance of repeat classes in the genome versus the Kimura divergence from the consensus.

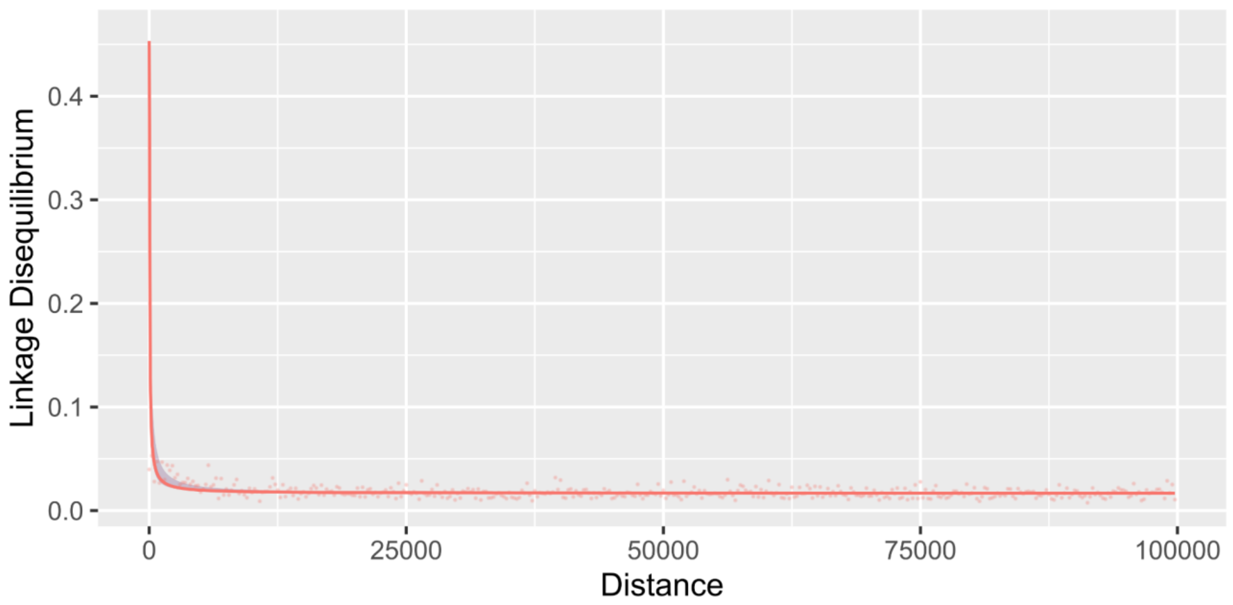

Fig S2. Fitting of r^2 decay curve of Linkage Disequilibrium. X-axis, the physical distance between loci; Y-axis, r^2 decay for linkage disequilibrium.

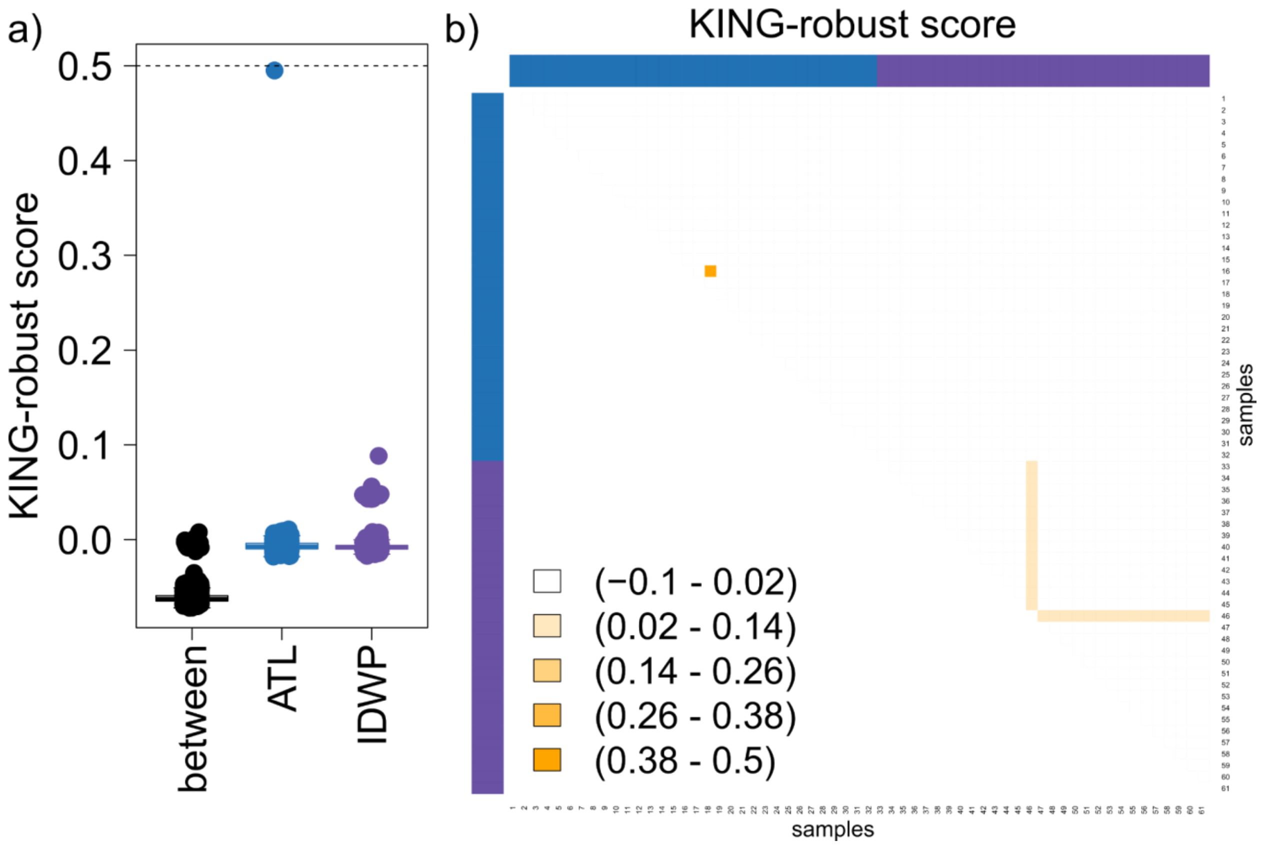

Fig S3. Relatedness inference estimated by the Kinship-based INference for Genome-wide association studies (KING). a) Within and between populations. b) Between individuals. Yellow scale: KING-robust scores. ATL, Atlantic population: IDWP, Indo-Western Pacific population.

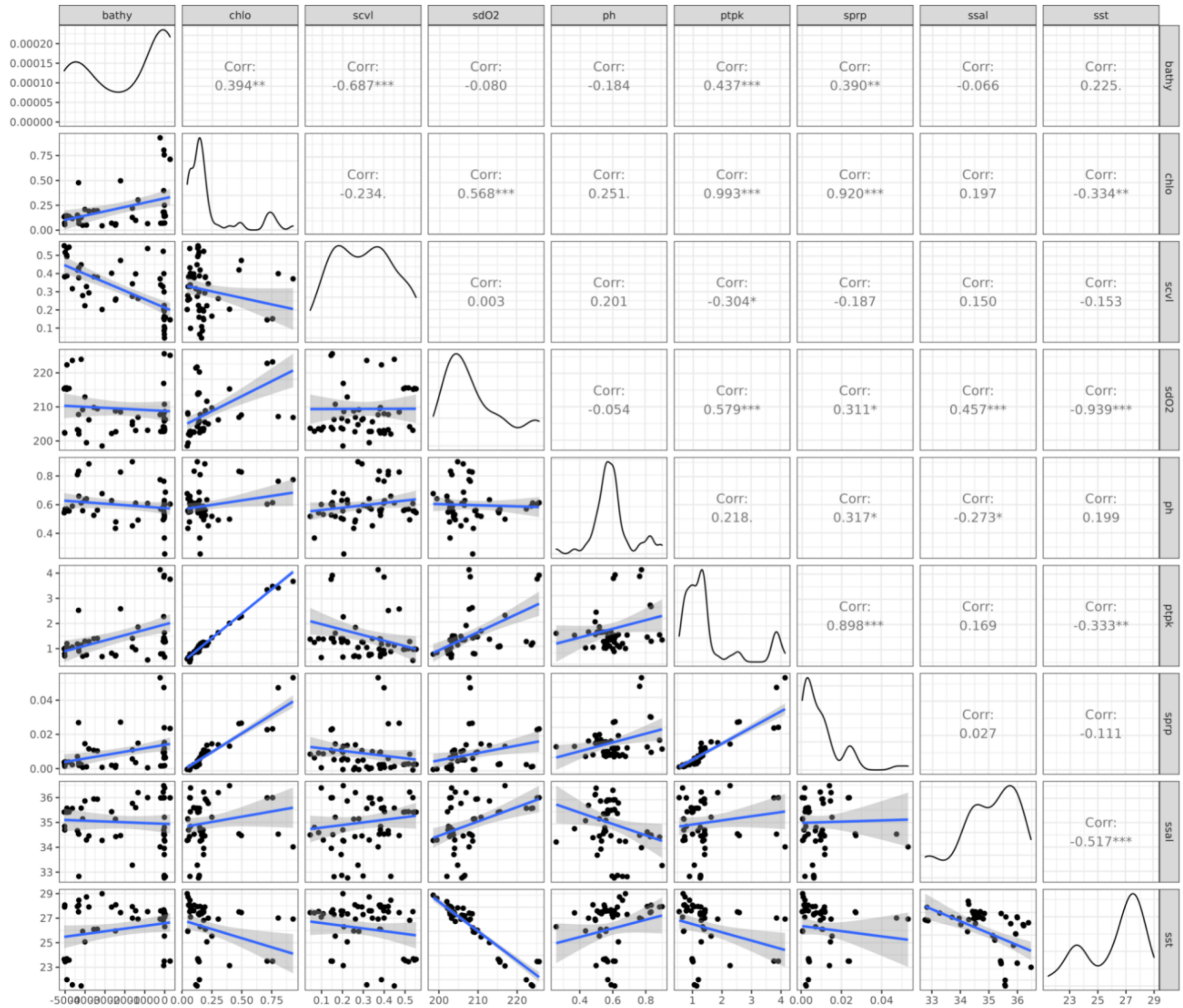

Fig S4. Correlation matrix of all tested environmental variables. Scatterplots of each pair of the numeric variables are drawn on the left. Pearson correlation is displayed on the right. Variable distribution is available on the diagonal. bathy, mean bathymetry; chlo, mean concentration of surface chlorophyll; scvl, mean velocity of surface marine currents; sdO2, mean concentration of surface dissolved molecular oxygen; pH, pH estimations; ptpk, mean concentration of surface phytoplankton; sprp, daily mean of primary surface productivity; ssal, mean concentration of surface salinity; sst, mean sea surface temperature. *** $p < 0.001$, ** $p < 0.01$, * $p < 0.05$.

a)

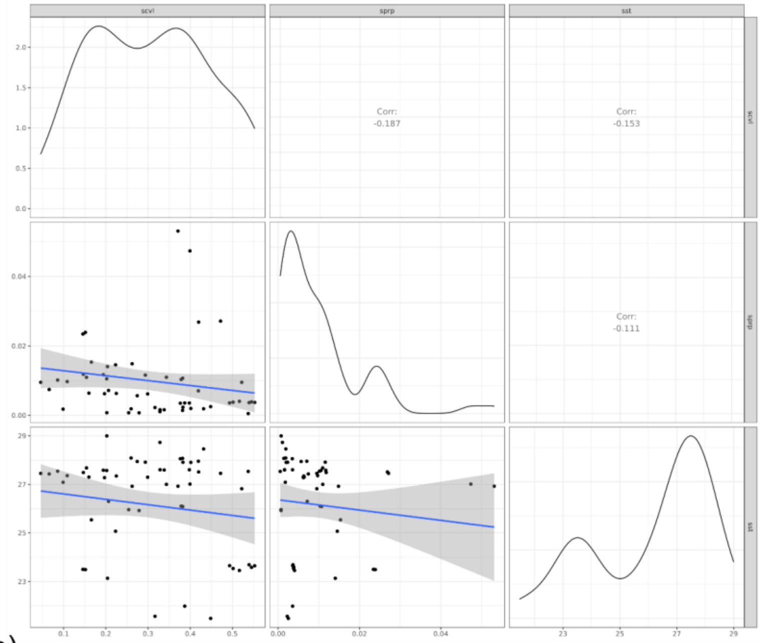

b)

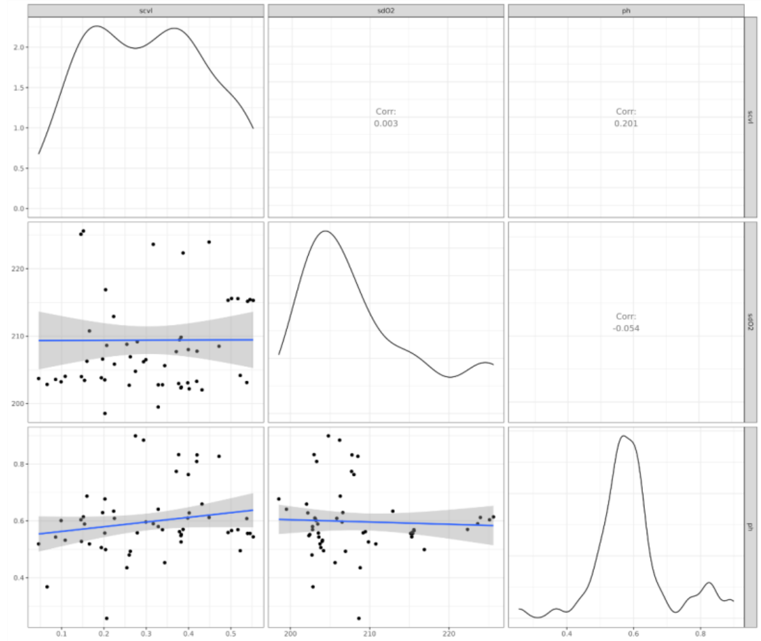

Fig S5. Correlation matrix of environmental variables without collinearity. Scatterplots of each pair of the numeric variables are drawn on the left. Pearson correlation is displayed on the right. Variable distribution is available on the diagonal. a) scvl, mean velocity of surface marine currents; sprp, daily mean of primary surface productivity; sst, mean sea surface temperature. b) scvl, mean velocity of surface marine currents; sdO2, mean concentration of surface dissolved molecular oxygen; pH, pH estimations. *** $p < 0.001$, ** $p < 0.01$, * $p < 0.05$.

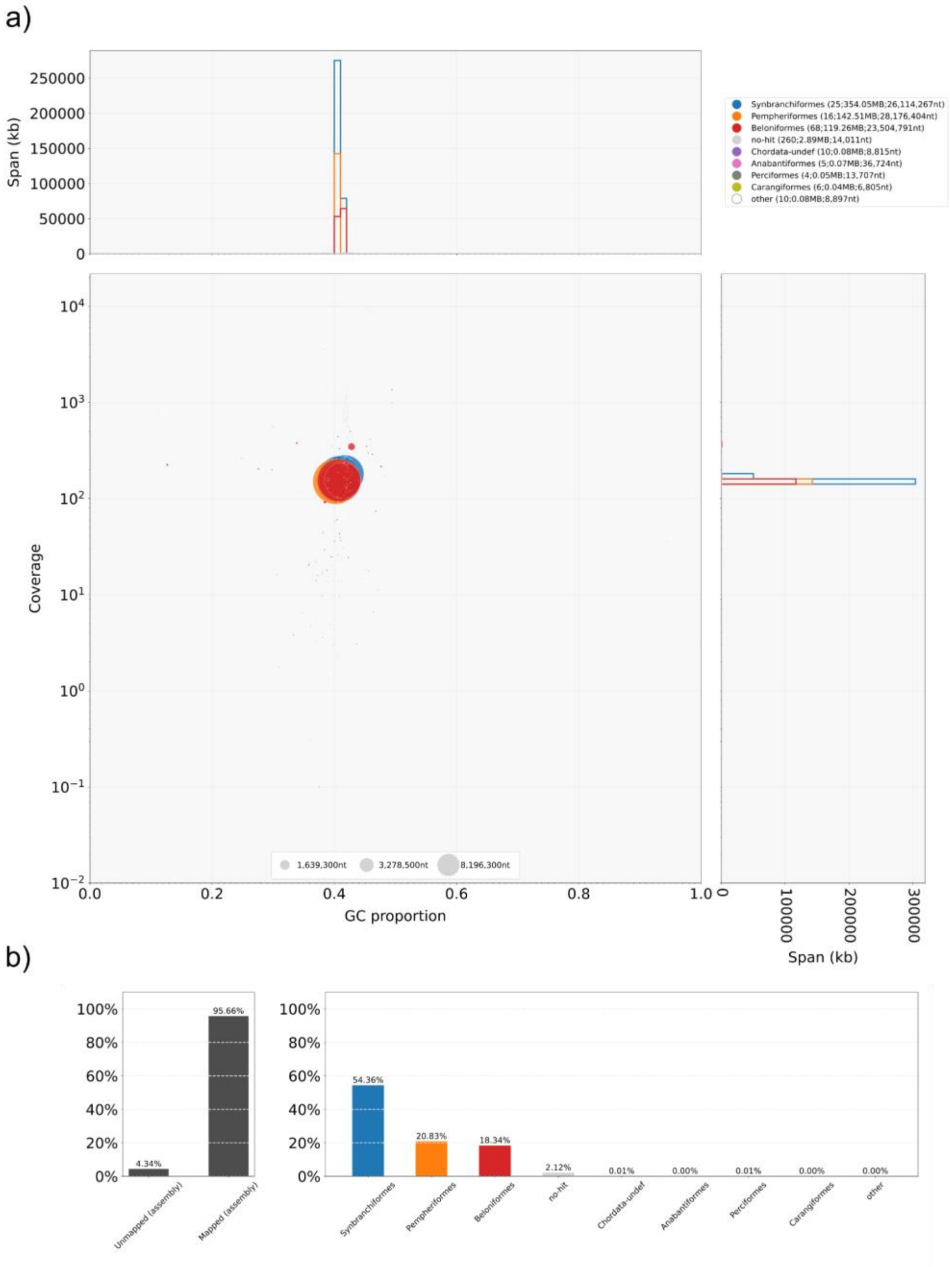

Fig S6. a) Blobtools plot with taxonomic assignments per scaffold resulting from a BlastN comparison against the NCBI nucleotide database and scaffold-wide GC content and coverage. Circles are positioned on the Y-axis in the scatter plot based on the base coverage of the sequence in the coverage library, a proxy for the molarity of input DNA in the sequencing reaction. Circles' position on the X-axis based on their GC content and the proportion of G and C bases in the sequence can differ between genomes. Coverage and GC histograms are drawn for each taxonomic group, weighted by the total span (cumulative length) of sequences occupying each bin. The results suggest an assembly with no noticeable contamination. b) ReadCovPlots visualizing the proportion of reads of a library that are unmapped or mapped, showing the percentage of mapped reads by taxonomic group.

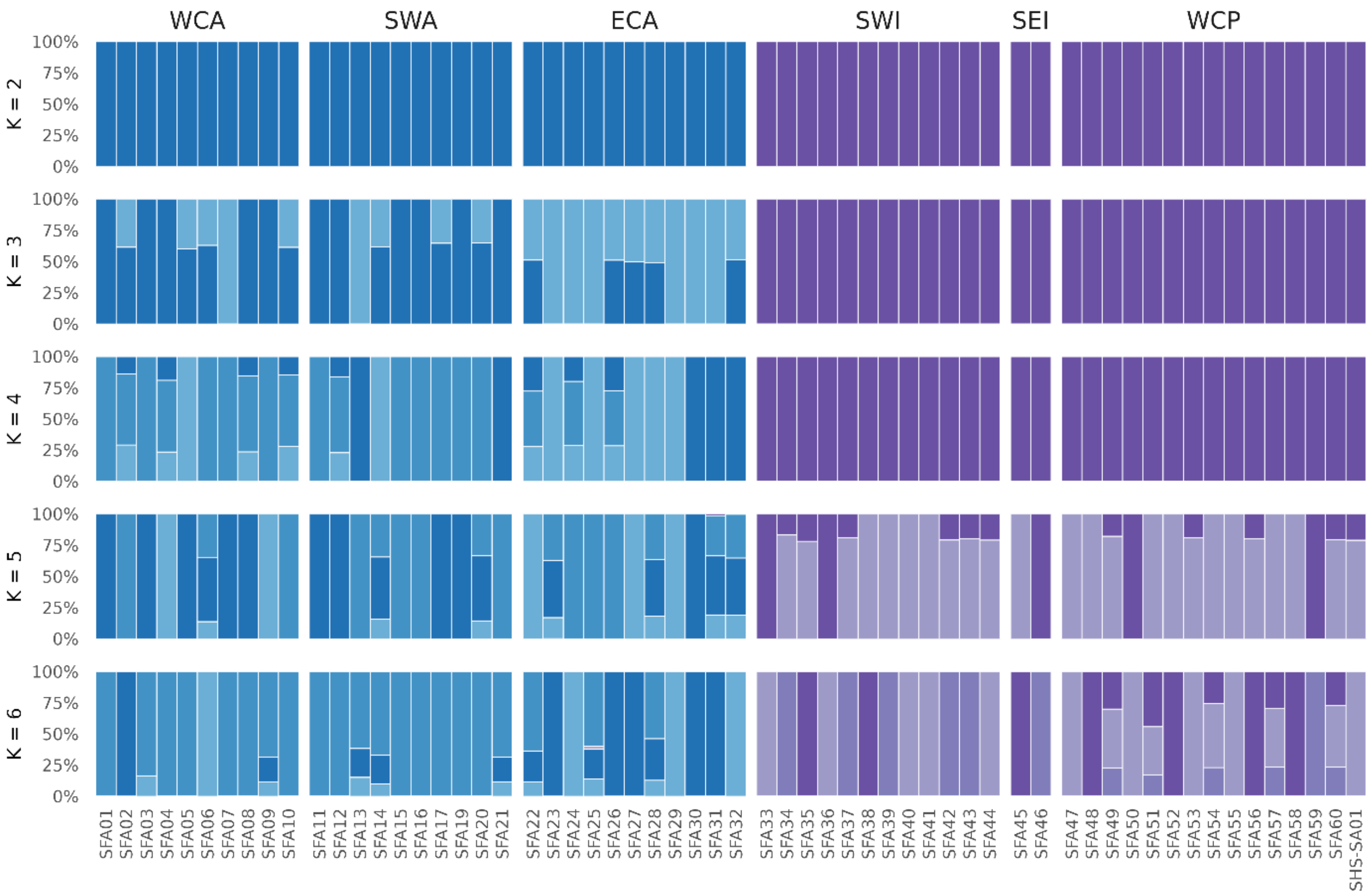

Fig S7. Individual ancestral admixture proportions estimations from K 2 to 6. WCA, Western Central Atlantic; SWA, Southwest Atlantic; ECA, Eastern Central Atlantic; SWI, Southwest Indian; SEI, Southeast Indian; WCP, Western Central Pacific. Colors represent the different clusters. K is the number of clusters.

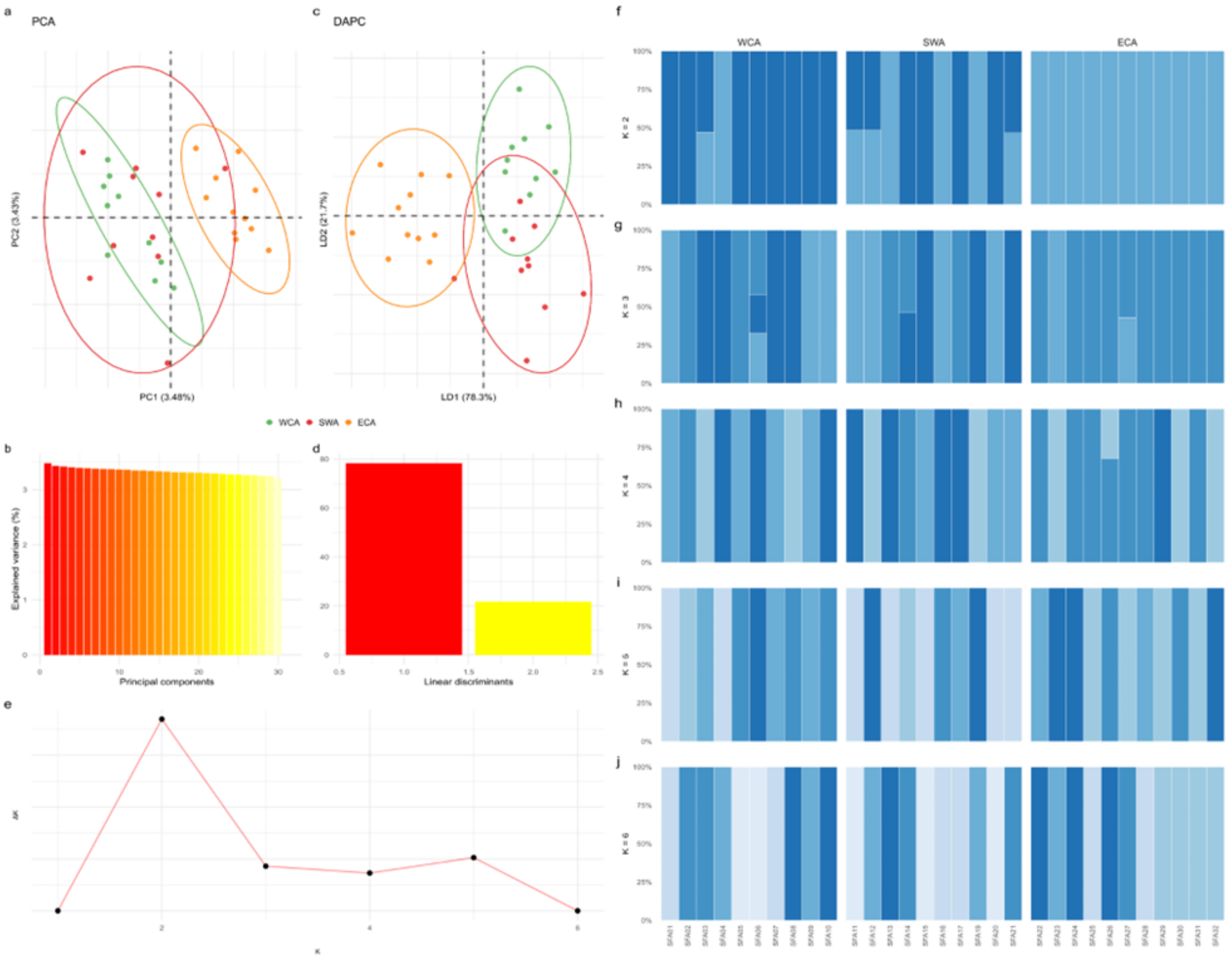

Fig S8. Atlantic Ocean clustering and admixture analysis. a) Principal Component Analysis (PCA) from principal coordinates 1 and 2. b) Eigenvalues from PCA. c) Discriminant Analysis of Principal Components (DAPC) from Discriminant Analysis (DA) 1 and 2. d) Eigenvalues for the DAs. e) Evanno's ΔK method. f–j) Individual ancestral admixture proportions estimations from K = 2 to 6, each color represents a genetic cluster. WCA, Western Central Atlantic; SWA, Southwest Atlantic; ECA, Eastern Central Atlantic.

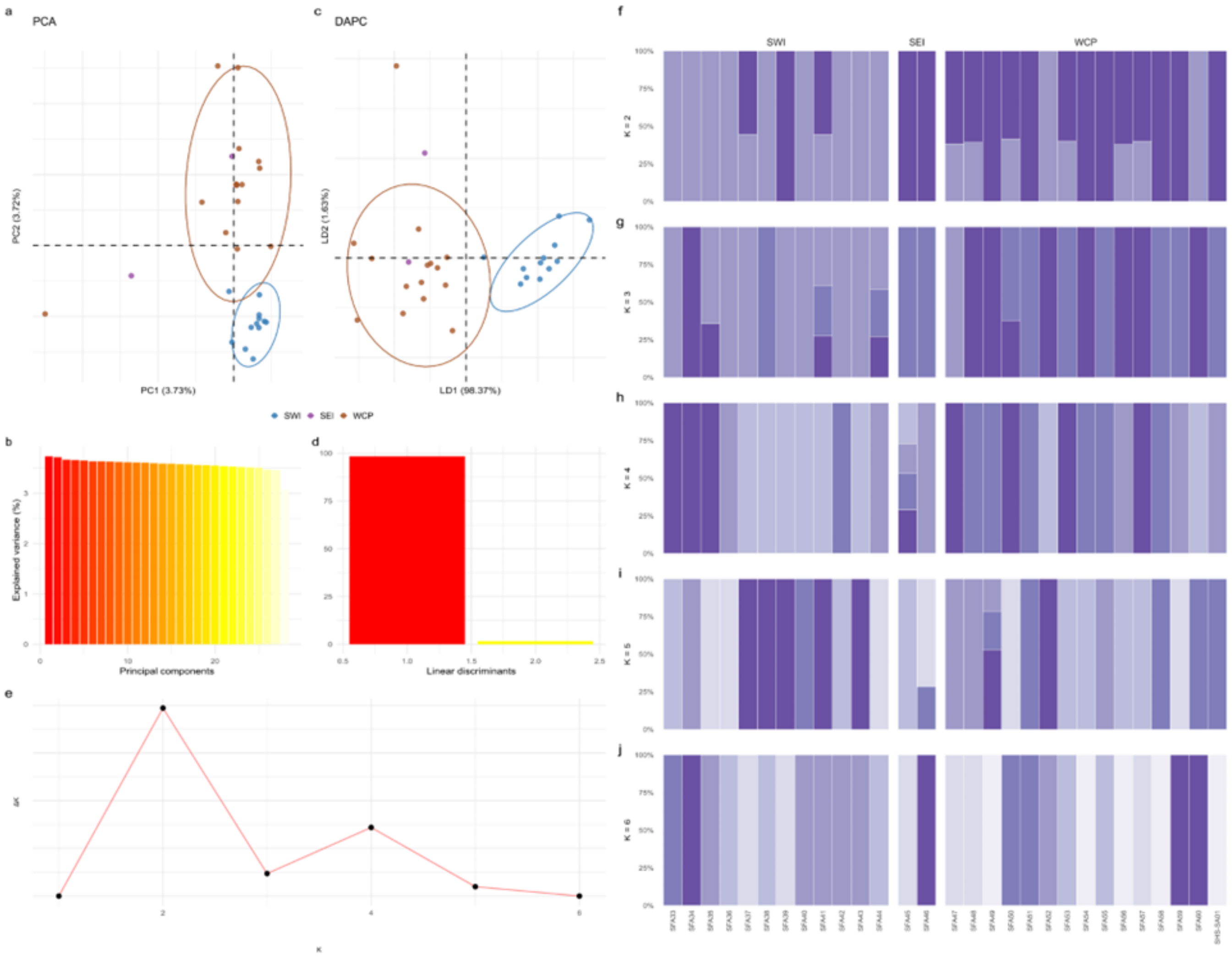

Fig S9. Indo-Western Pacific Ocean clustering and admixture analysis. a) Principal Component Analysis (PCA) from principal coordinates 1 and 2. b) Eigenvalues from PCA. c) Discriminant Analysis of Principal Components (DAPC) from Discriminant Analysis (DA) 1 and 2. d) Eigenvalues for the DAs. e) Evanno's ΔK method. f–j) Individual ancestral admixture proportions estimations from K = 2 to 6, each color represents a genetic cluster. SWI, Southwest Indian; SEI, Southeast Indian; WCP, Western Central Pacific.

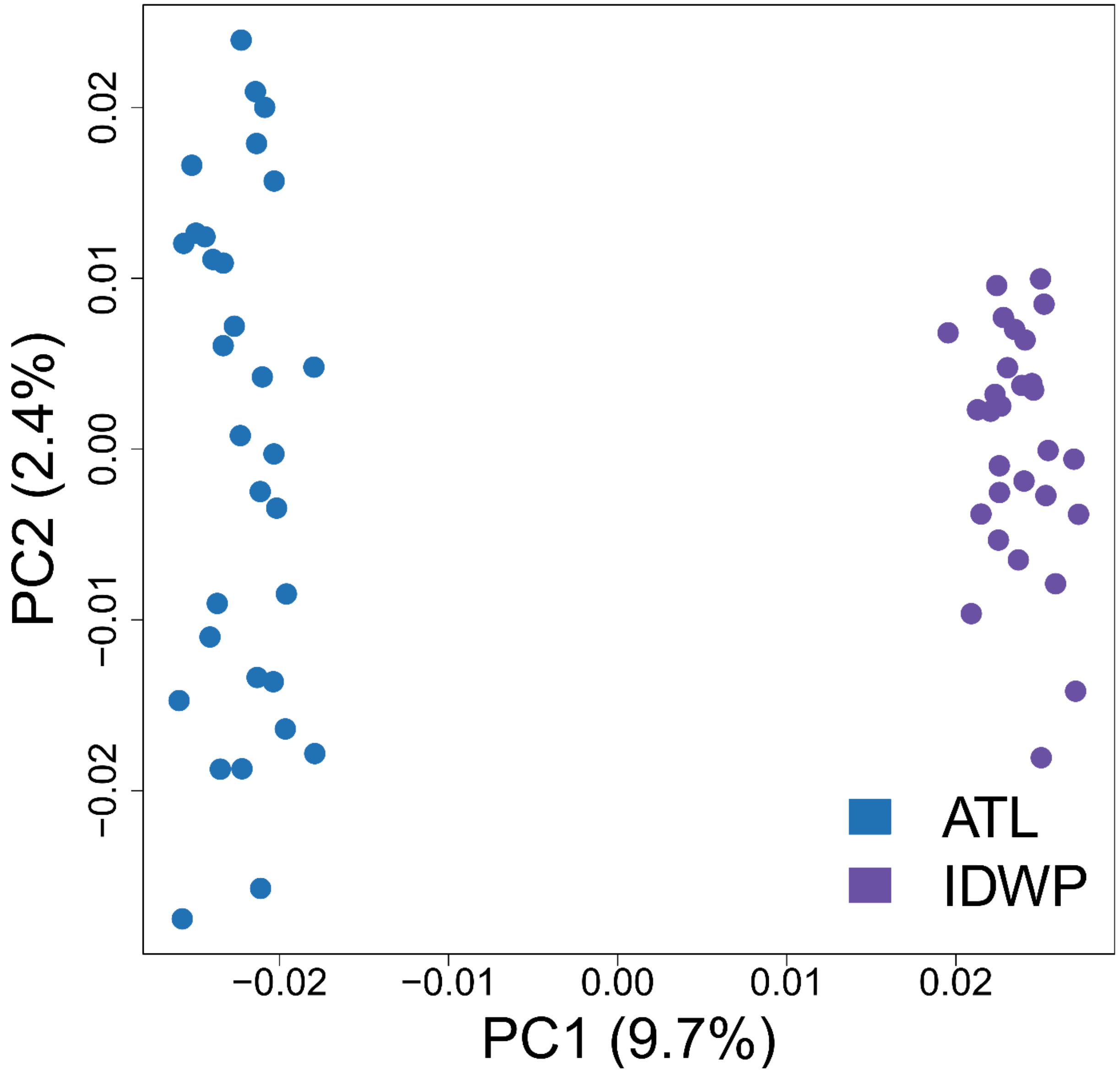

Fig S10. Principal Component Analysis (PCA) from principal coordinates 1 and 2 of the 200 highly informative SNPs subset.

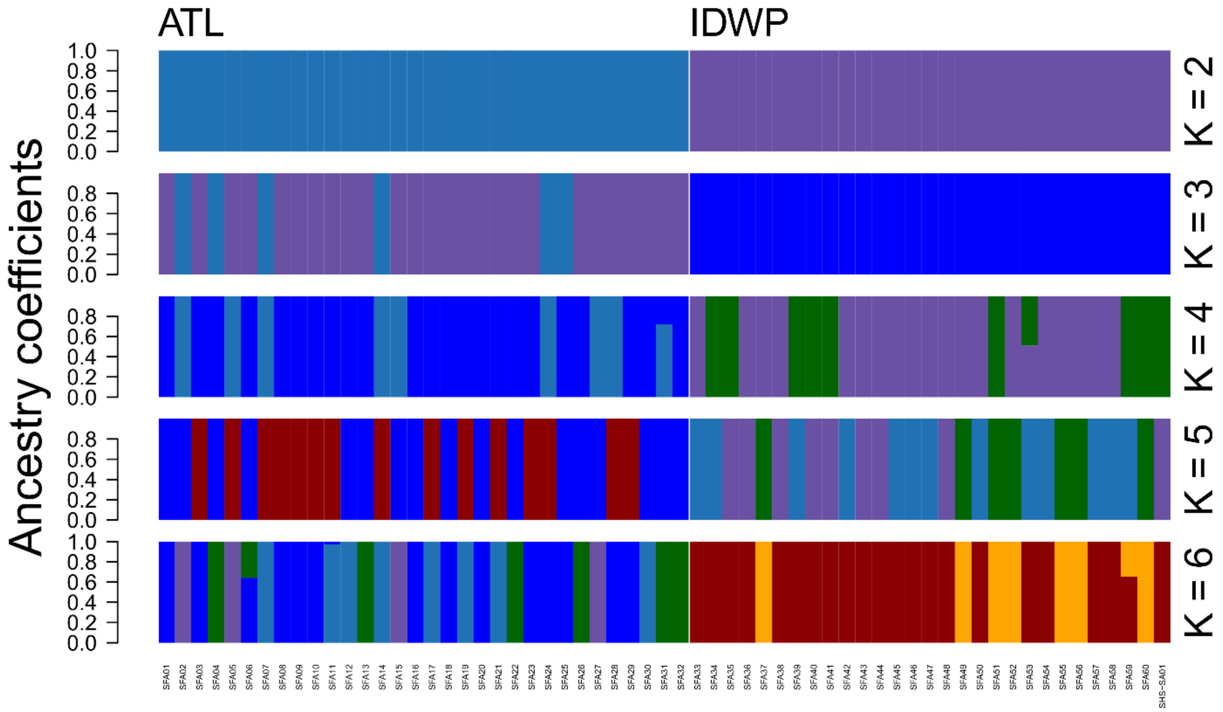

Fig S11. Individual ancestral admixture proportions estimations from K = 2 to K = 6, each color represents a genetic cluster of the 200 highly informative SNPs subset.

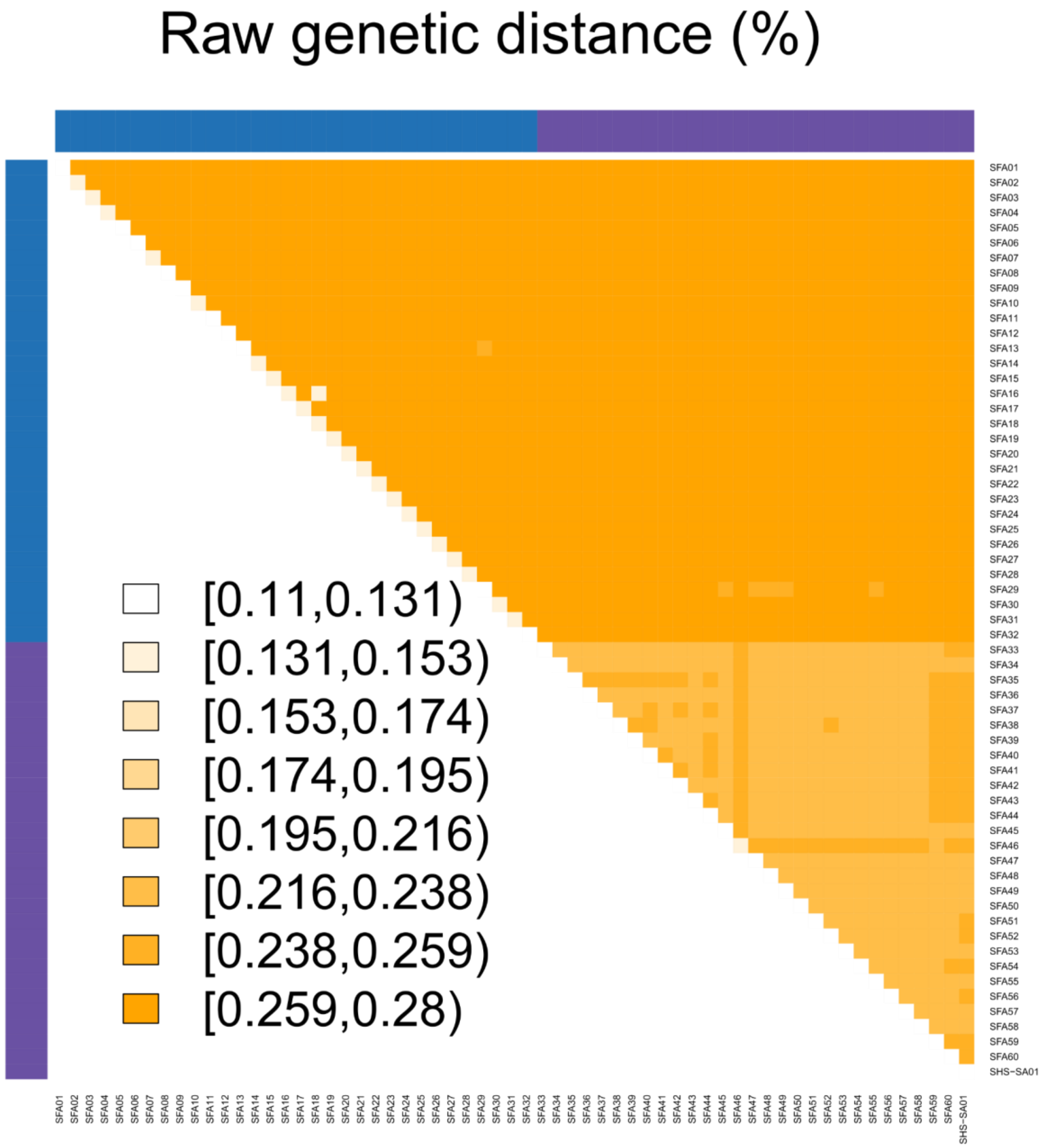

Fig S12. Raw genetic distance between genotypes. Because the data was unphased genotypic data, the raw genetic distance between two individuals could not be measured directly but was estimated as the expected distance between two randomly drawn haplotypes. Sites were categorized in four categories: XX-XX, XX-XY, XY-XY, and XX-YY, in which X and Y represent genotype scores of individual 1 and individual 2 for a given site. The total number of sites for each of the four categories was denoted by: n0, n1, n2he, and n2ho. Raw genetic distance, in percentages, was calculated using the formula: d = (n1*0.5+n2he*0.5+n2ho)/(n0+n1+n2he+n2ho)*100 in percentages.

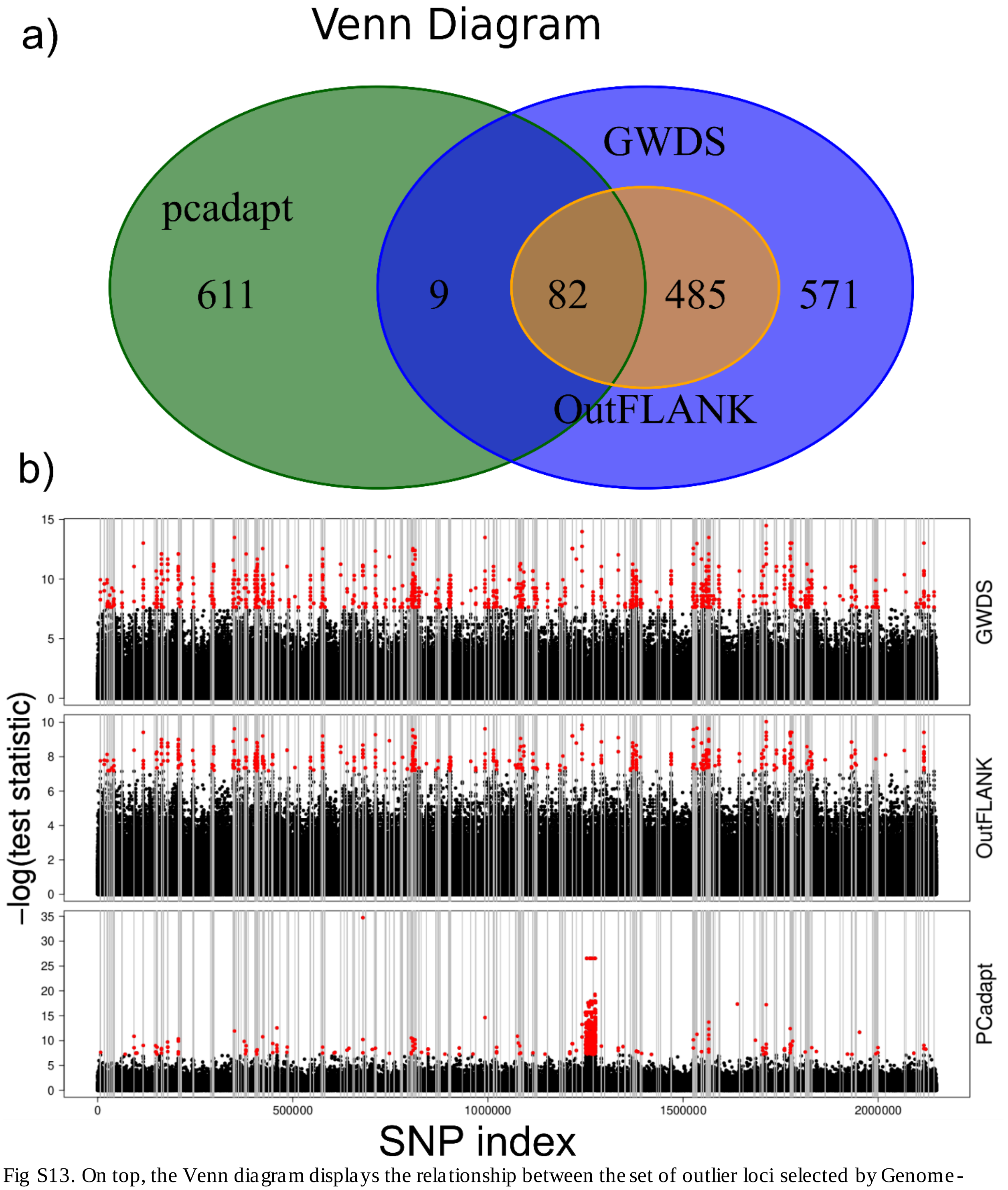

Fig S13. On top, the Venn diagram displays the relationship between the set of outlier loci selected by Genome-Wide Differentiation Scan (GWDS), pcadapt, and OutFLANK. Below are the Manhattan plots of each genome scan.

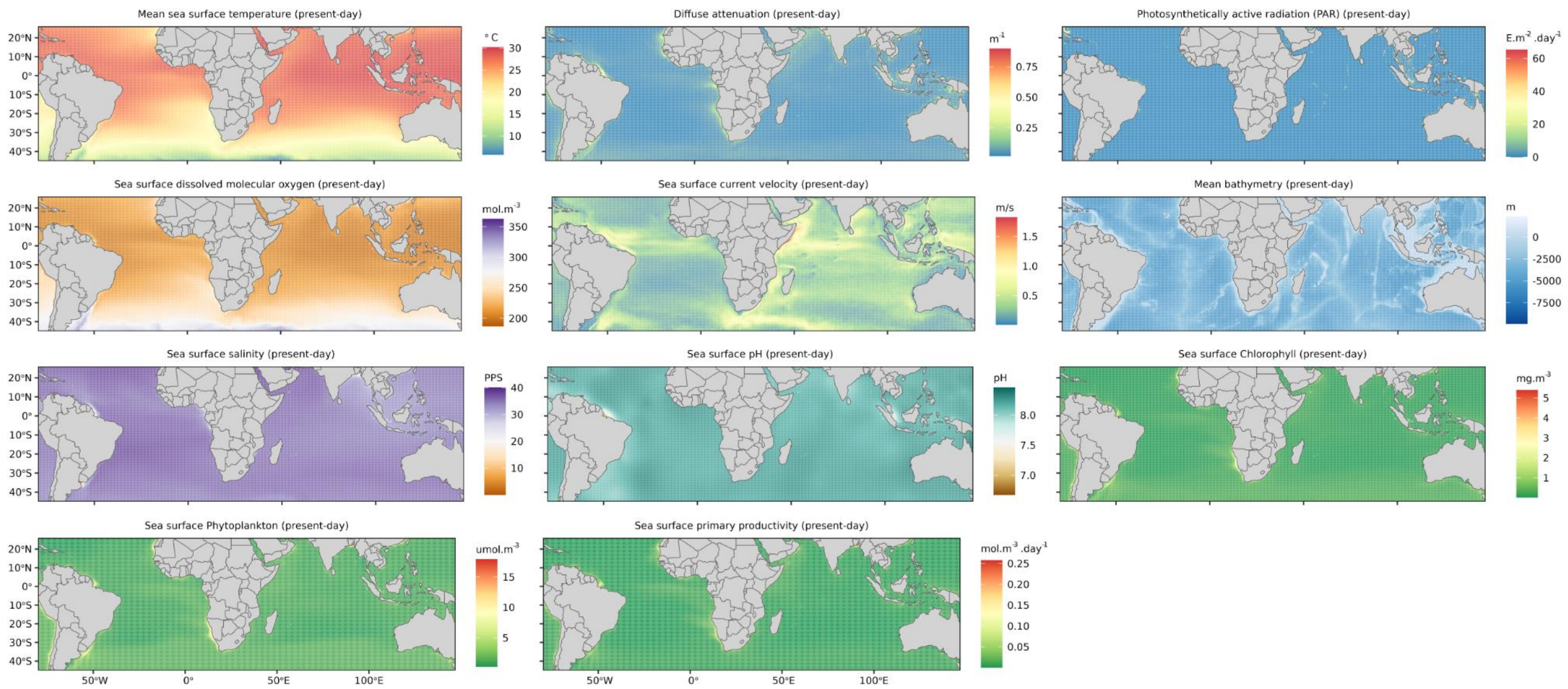

Fig S14. Heatmaps of the tested environmental variables. a) mean sea surface temperature. b) mean concentration of surface dissolved molecular oxygen. c) mean velocity of surface marine currents. d) mean bathymetry. e) mean concentration of surface salinity. f) pH estimations. g) mean concentration of surface chlorophyll. h) mean concentration of surface phytoplankton: i) daily mean of primary surface productivity.

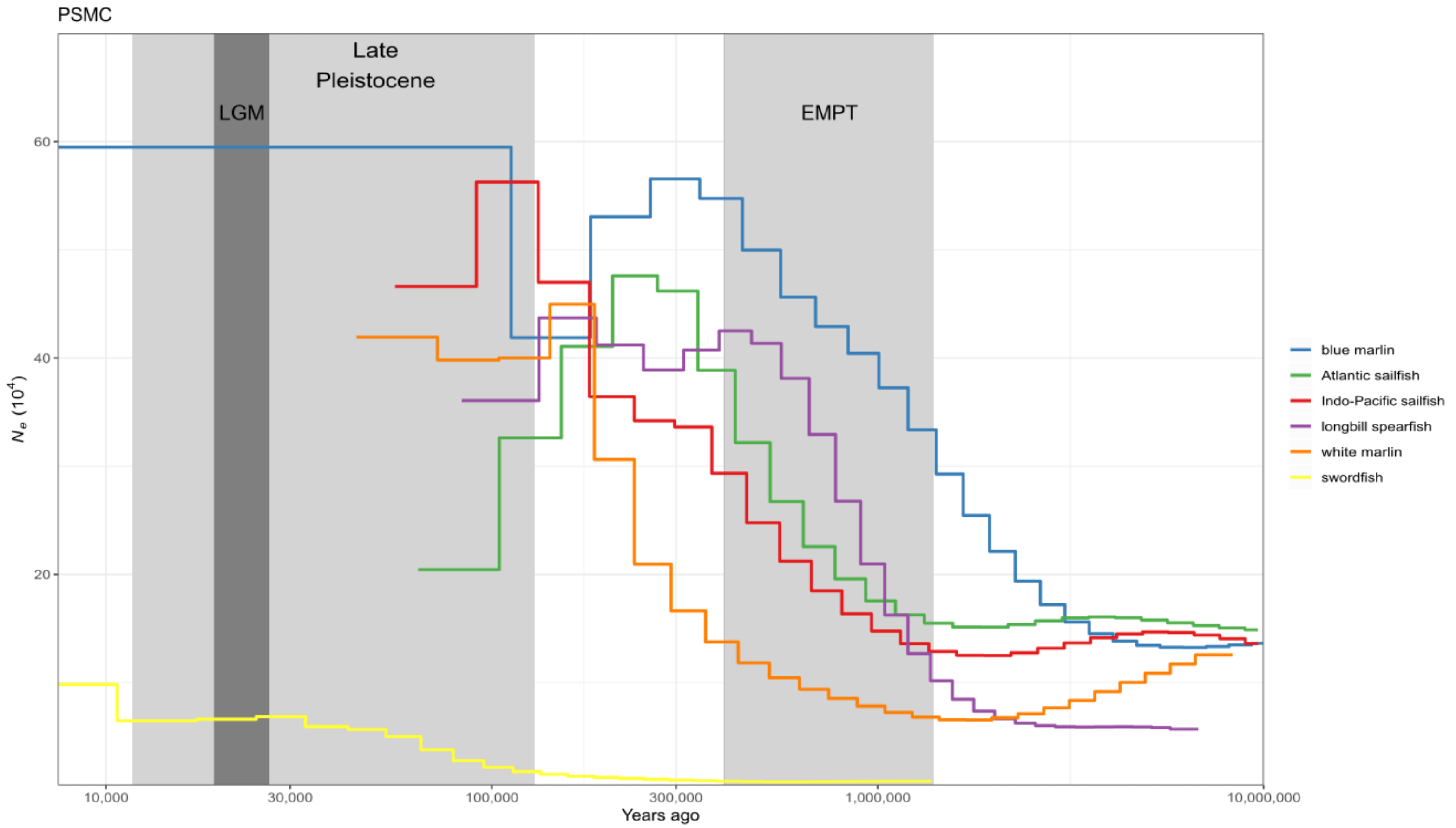

Fig S15. Pairwise Sequentially Markovian Coalescent (PSMC) of billfishes. N_e: effective population size; LGM: Last Glacial Maximum; EMPT: Early Middle Pleistocene Transition.

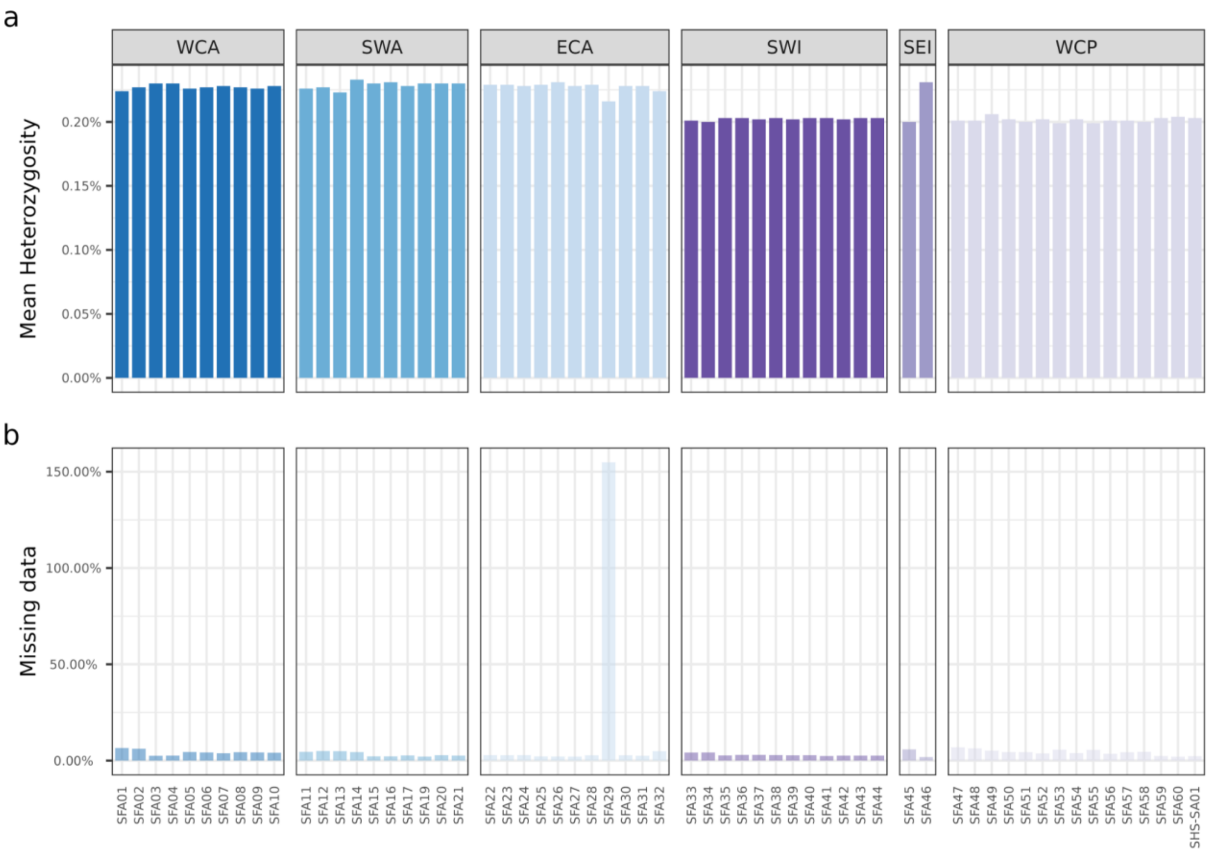

Fig S16. Genome-wide heterozygosity and missing data frequency per individual. WCA, Western Central Atlantic; SWA, Southwest Atlantic; ECA, Eastern Central Atlantic; SWI, Southwest Indian; SEI, Southeast Indian; WCP, Western Central Pacific.

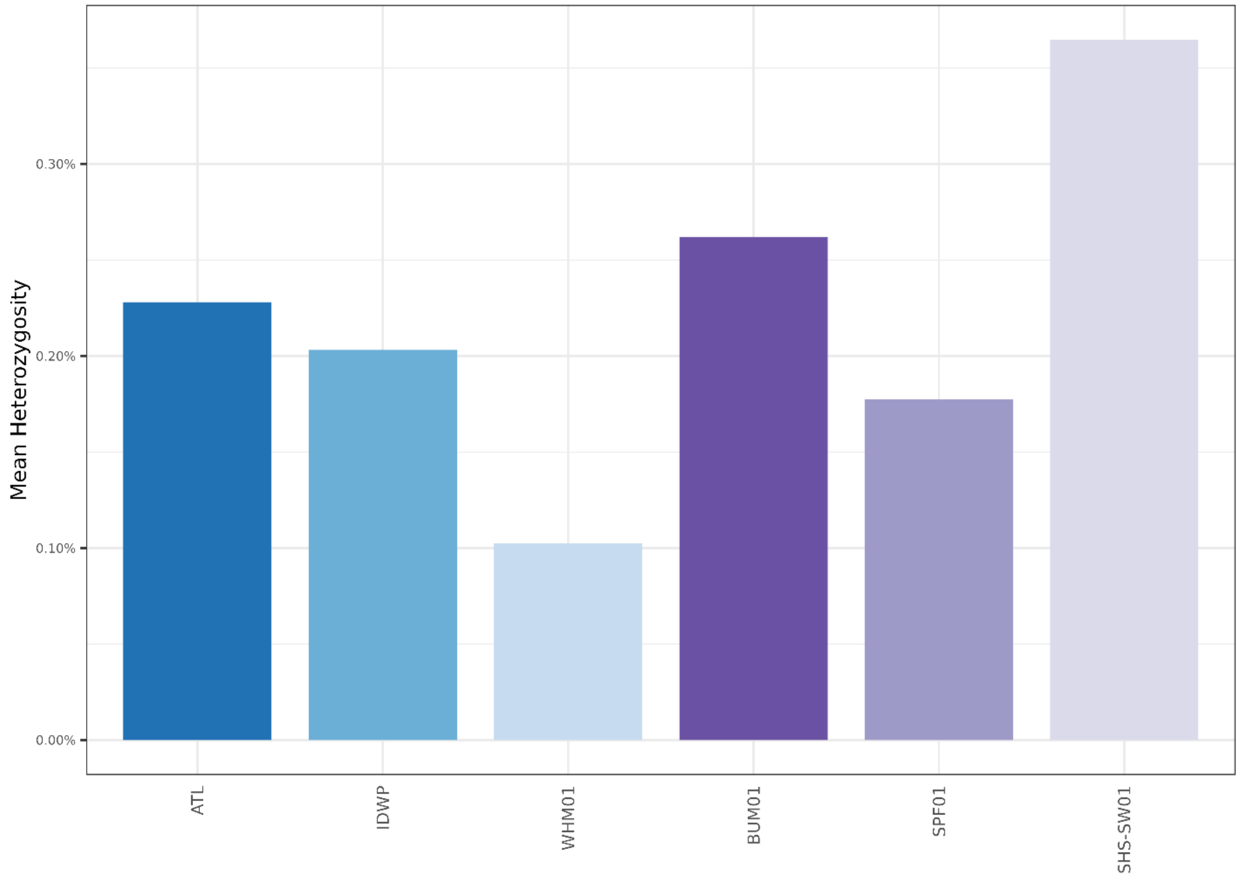

Fig S17. Genome-wide heterozygosity among billfishes. ATL, Atlantic sailfish population; IDWP, Indo-Western Pacific sailfish population; BUM, *Makaira nigricans*; WHM, *Kajikia albida*; SPF, *Tetrapturus angustirostris*, SHS-SW01, *Xiphias gladius*.

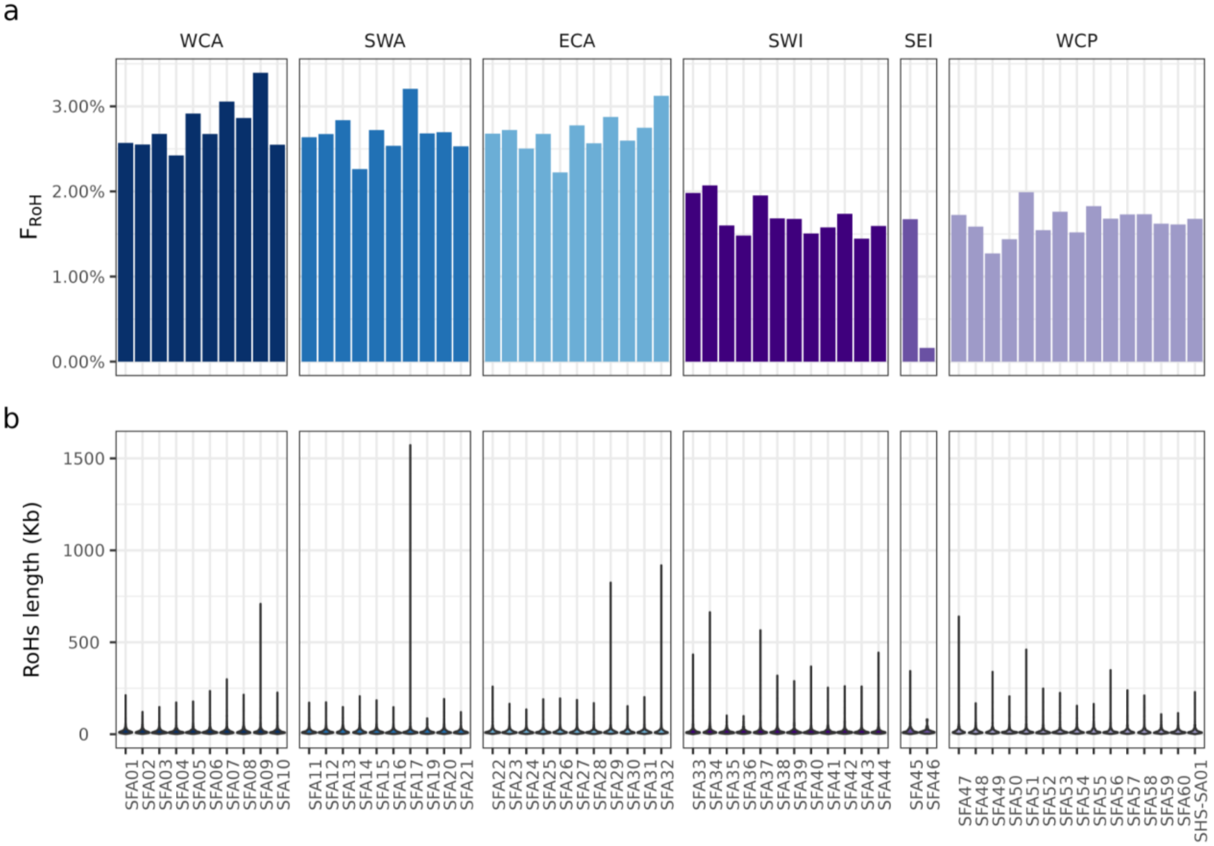

Fig S18. Runs of Homozygosity (RoHs) per individual. WCA, Western Central Atlantic; SWA, Southwest Atlantic; ECA, Eastern Central Atlantic; SWI, Southwest Indian; SEI, Southeast Indian; WCP, Western Central Pacific.

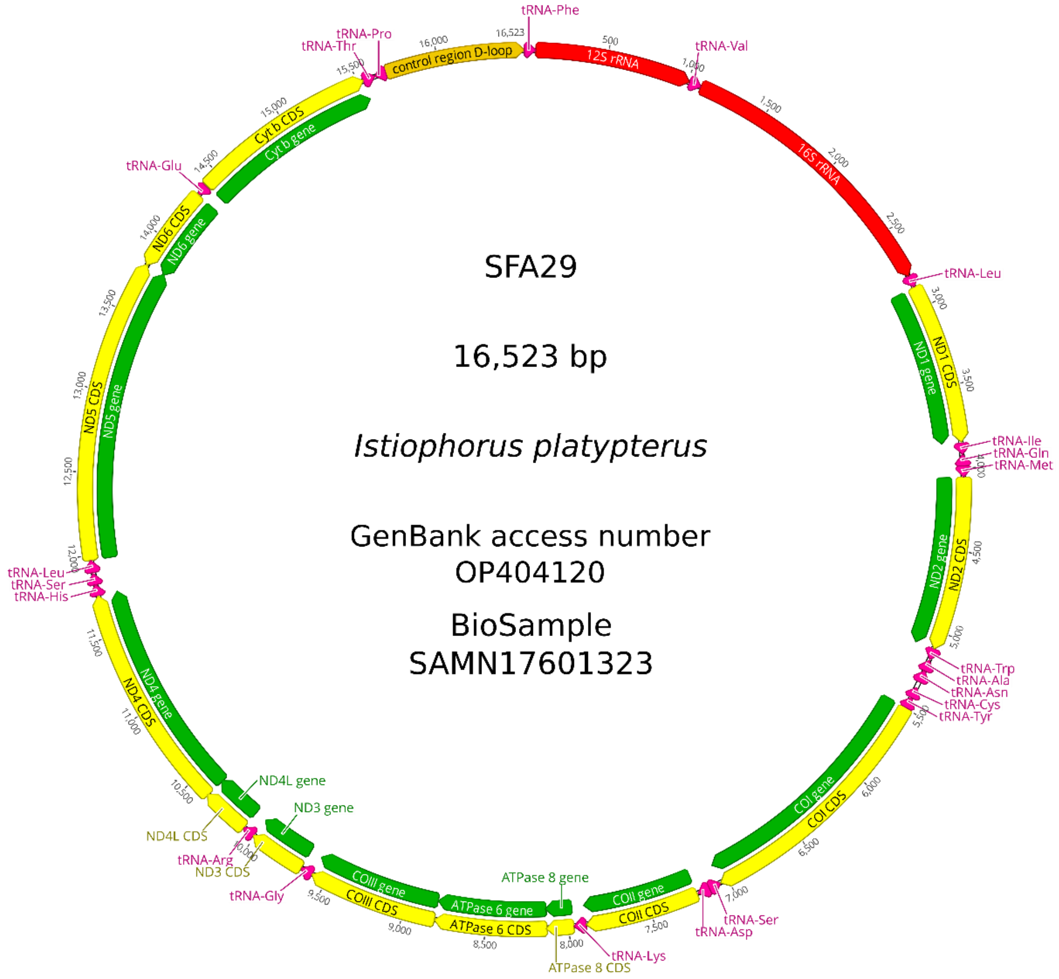

Fig S19. Representation of an annotated mitogenome of the sailfish (*Istiophorus platypterus*).

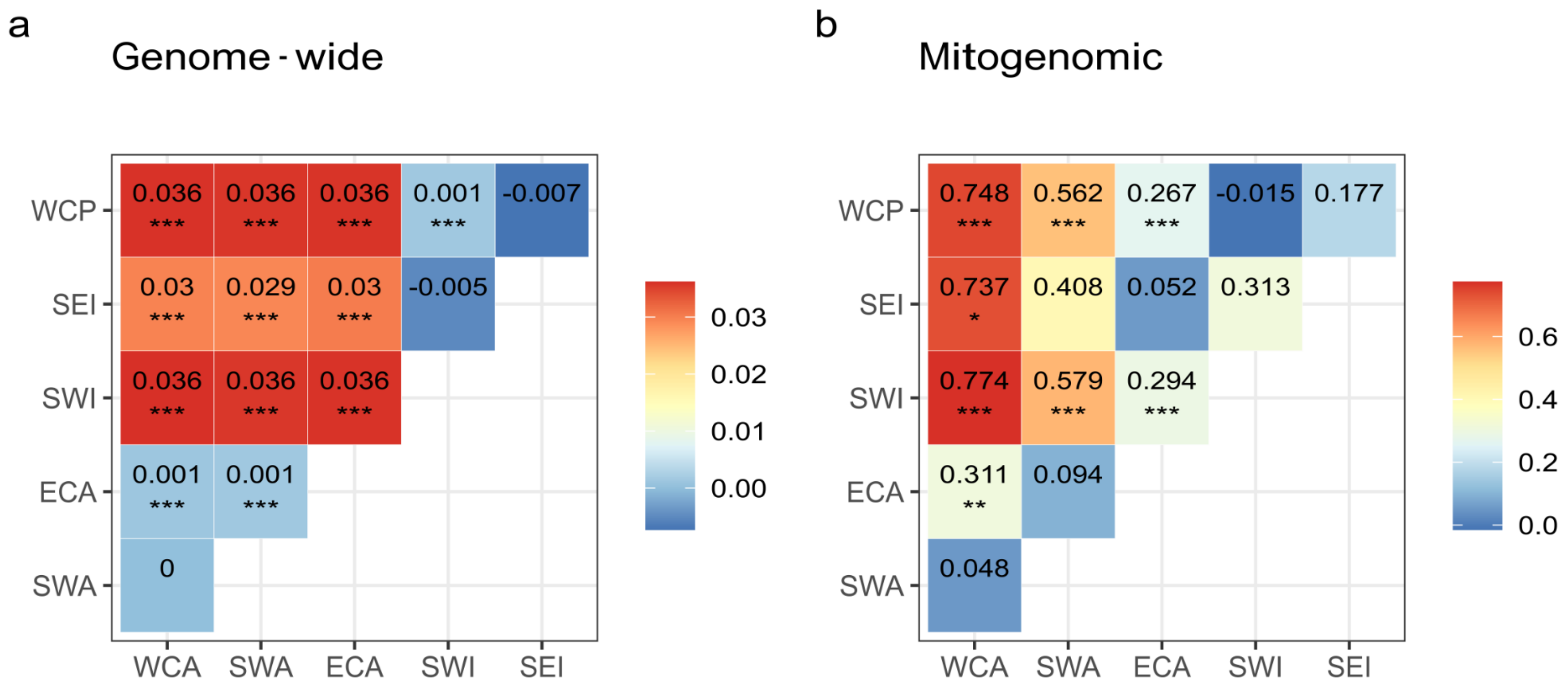

Fig S20. a) Genome-wide Pairwise F_{ST} of LD pruned SNPs. b) Pairwise Φ_{ST} of mitogenomic haplotypes. WCA, Western Central Atlantic; SWA, Southwest Atlantic; ECA, Eastern Central Atlantic; SWI, Southwest Indian; SEI, Southeast Indian; WCP, Western Central Pacific. ***, $p < 0.0001$; **, $p < 0.001$; *, $p < 0.05$.

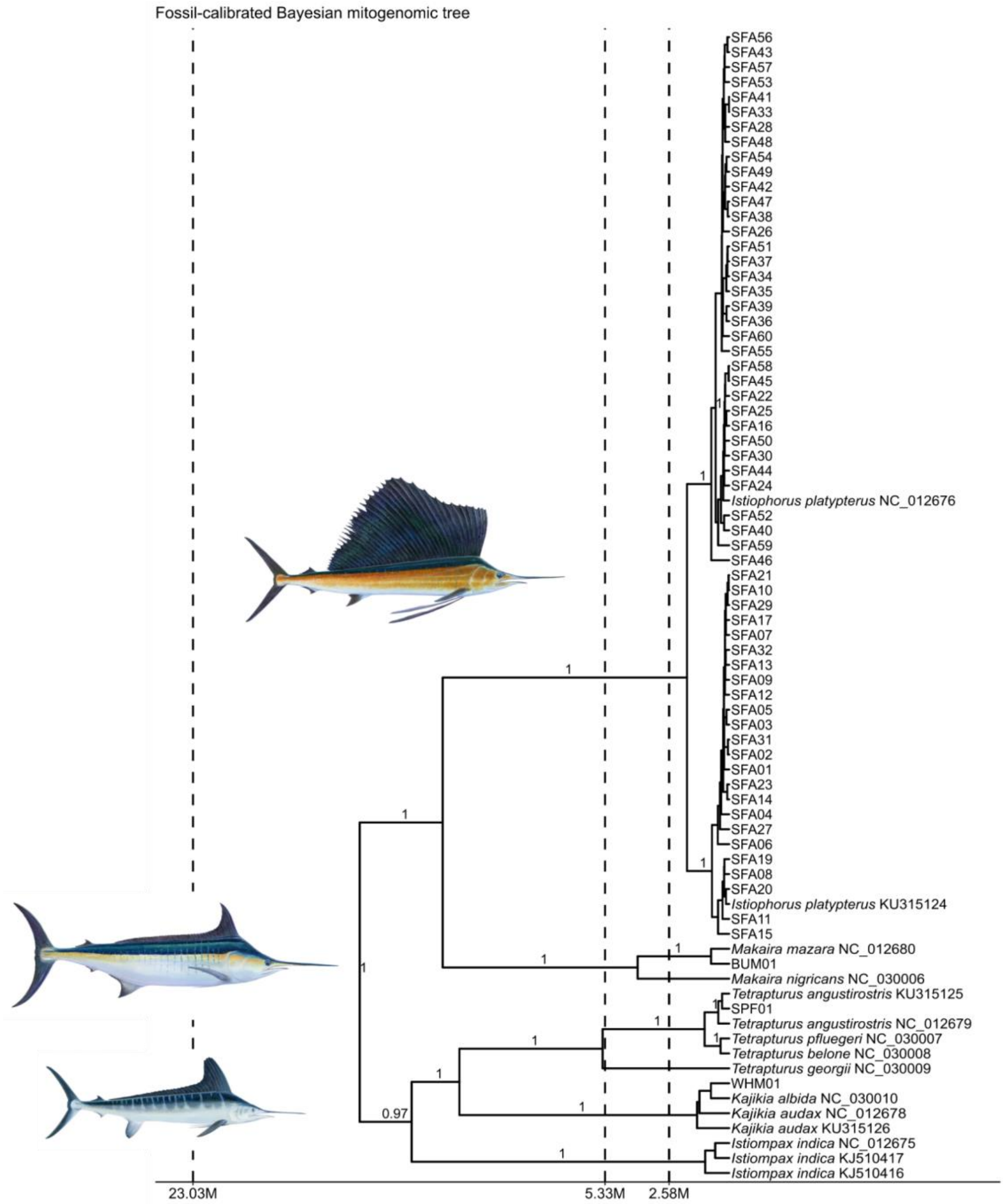

Fig S21. Fossil-calibrated Bayesian phylogenetic inference displaying the posterior probabilities support for each node. Dashed lines represent the lower boundaries between the Cenozoic epochs, Pleistocene (2.58M), Pliocene (5.33M), and Miocene (23.03M), respectively. SFA, *Istiophorus platypterus*; BUM, *Makaira nigricans*; WHM, *Kajikia albida*; SPF, *Tetrapturus angustirostris*.

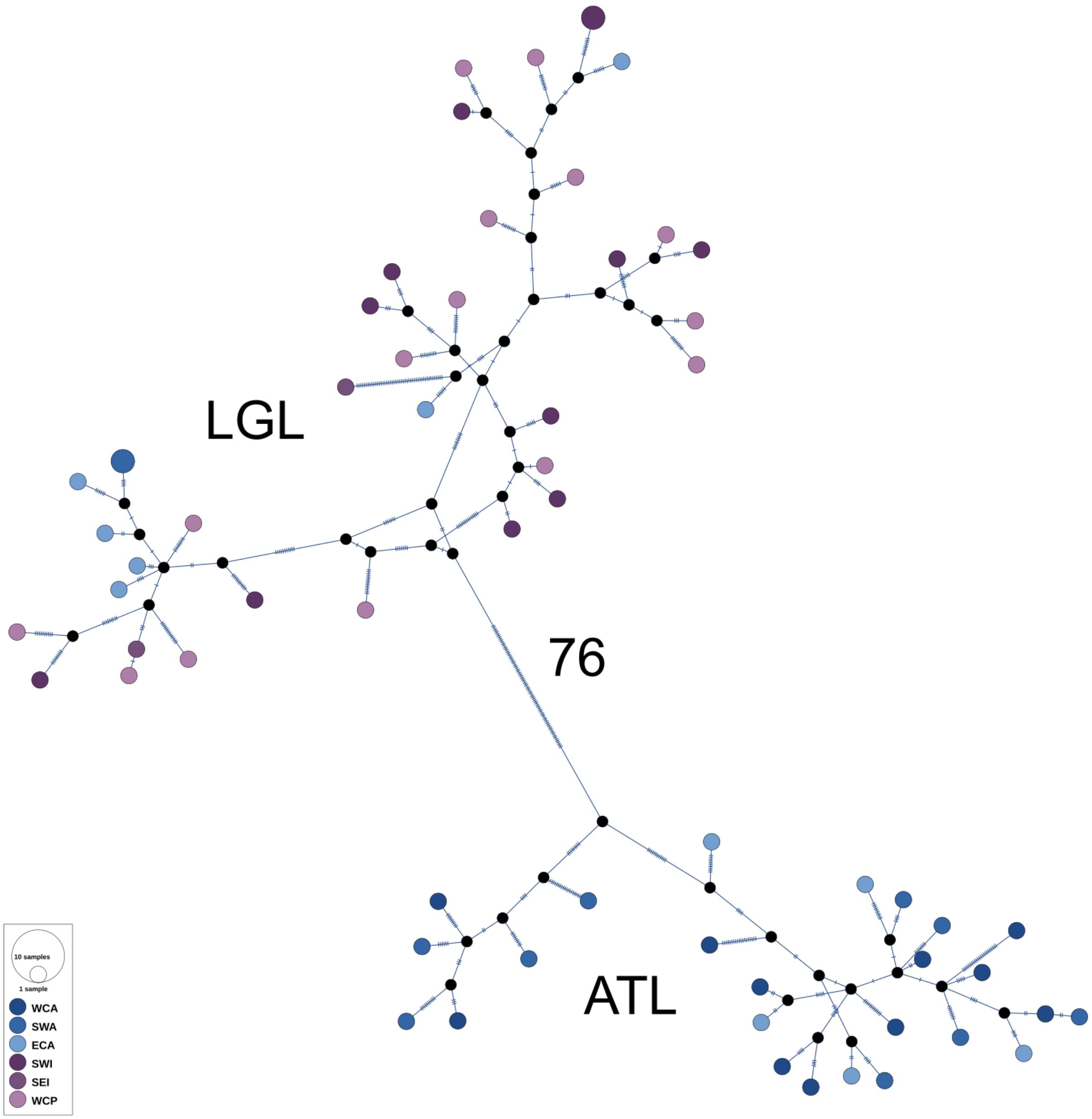

Fig S22. Mitogenomic TCS haplotype network represents genealogical relationships among haplotypes at the population level. Black dots are unsampled hypothetical haplotypes. Scratch marks represent the mutational steps between haplotypes. WCA, Western Central Atlantic; SWA, Southwest Atlantic; ECA, Eastern Central Atlantic; SWI, Southwest Indian; SEI, Southeast Indian; WCP, Western Central Pacific, LAT, Atlantic lineage; LGL, Global lineage.

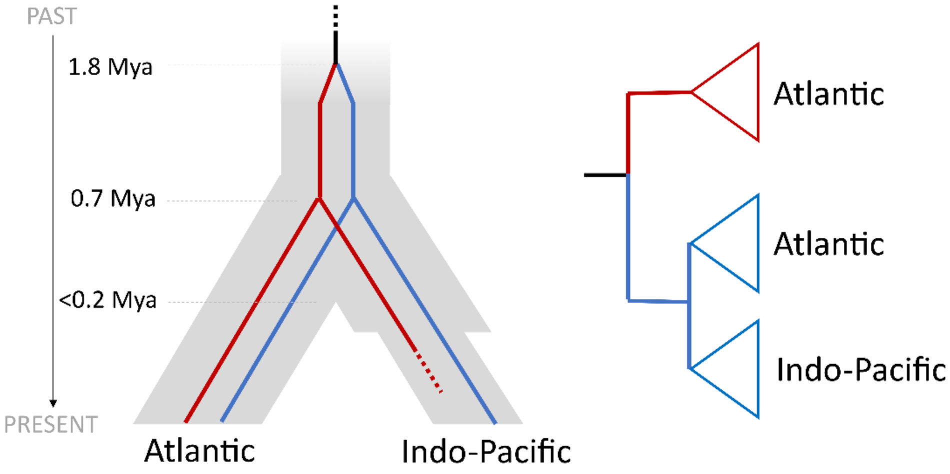

Figure S23. Incomplete Lineage Sorting (ILS) scenario of the mito-nuclear discordance.
